# Supplementary material for: Estimated impact of RTS,S/AS01 malaria vaccine allocation strategies in sub-Saharan Africa: A modelling study
Source: PLoS Med. 2020 Nov 30;17(11):e1003377. doi: 10.1371/journal.pmed.1003377 (PMC7703928; doi:10.1371/journal.pmed.1003377)
Supplement: S1 Appendix — This content includes Fig A and Tables A to D. (PDF) [file pmed.1003377.s001.pdf]

S1 Appendix: Mathematical model and parameters

We used an individual-based model of malaria transmission. Full details of the model and interventions other than the vaccine are published elsewhere [1]. The key details are reproduced here for completeness.

1.1 Transmission model

Individuals begin life susceptible to infection (*S*) but with a degree of maternal immunity (see below) that decays over their first six months. Susceptible individuals (i) experience a force of infection Λ_i which depends on the vectorial capacity at a given time as well as their level of pre-erythrocytic immunity (see below). Following infection, individuals experience a delay of length d_E representing the period from infection to appearance of blood-stage infection, following which they develop either symptomatic clinical disease or asymptomatic infection (*A*), with a probability dependent on their level of clinical immunity (see below). Symptomatic individuals receive appropriate treatment with probability f_T following which they enter the treated disease state (*T*) and otherwise enter the untreated disease state *D*. Those in the latter state eventually resolve symptoms and move to the asymptomatic state (*A*). Those that are treated subsequently experience a period of drug-dependent partial protection from re-infection (modelled as a Weibull survivorship function) before returning to the fully susceptible state (*S*). From the asymptomatic state, as parasite density gradually reduces due to the immune response, asymptomatic individuals move to the sub-patent infection state (U) after which they clear infection and return to the susceptible state *S*. Individuals in states *D*, *A* and *U* can be re-infected (super-infection) and will move into infection states *D*, *T* or *A* following the same process as for primary infection. The flow between states is summarised in Fig A and the corresponding transitions in Table A.

Deaths from non-malaria associated causes are modelled using national life-tables [2], with individuals removed from the population at age-specific rates to match the required age distribution. Malaria-associated deaths are tracked separately (see below). When an individual dies they are replaced with a newborn individual with the same characteristics (heterogeneity in biting rates – see below) so that the population size in the simulation remains constant.

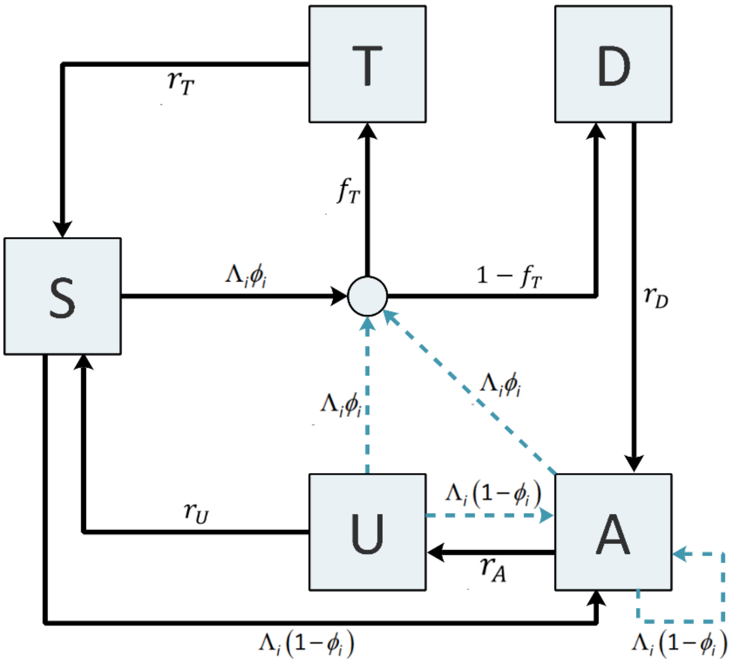

Fig A: Progression between human infection states. States are shown in boxes and state transitions by arrows with associated hazard rates. The circle represents the treatment node. Superinfection is indicated by dashed blue arrows. *S* = susceptible, *D* = clinical disease, *T* = successfully treated disease, *A* = asymptomatic patent infection, *U* = asymptomatic sub-patent infection. Malaria-associated deaths are tracked separately. Diagram is reproduced from S1 Appendix to Winskill et al. [1].

Table A: Infection state transition rates for the human component of the transmission model.

Process	Transition	Rate
Infection	$S \rightarrow I$	$\Lambda_i(t-d_E)$
Progression of untreated disease to asymptomatic infection	$D \rightarrow A$	$r_D = \frac{1}{d_D}$
Progression of asymptomatic to sub-patent infection	$A \rightarrow U$	$r_A = \frac{1}{d_A}$
Progression of sub-patent infection to susceptible	$U \rightarrow S$	$r_U = \frac{1}{d_U}$
Progression of treated disease to susceptible*	$T \rightarrow S$	$r_T = \frac{1}{d_T}$
Super-infection from untreated clinical disease, asymptomatic or sub-patent infection	$D \rightarrow I$<br>$A \rightarrow I$<br>$U \rightarrow I$	$\Lambda_i(t-d_E)$

* Treated individuals experience a period of drug-dependent partial protection from reinfection.

1.1.1 Heterogeneity in biting rates

Each individual in the simulation experiences a biting rate that depends on the product of their age-dependent biting rate, $\psi(a)$, and their relative biting rate ζ_i. For an individual of age *a* the former is defined as:

$$\psi(a) = 1 - \rho \exp\left(-\frac{a}{a_0}\right) \quad (1)$$

where ρ and a_0 are parameters that determine the relationship between age (i.e. body size) and biting rate and ω is a normalising constant for the biting rate with age

$$\omega = \int_0^\infty \psi(a) g(a)\ da \quad (2)$$

and $g(a)$ is the cross-sectional human population age distribution. The relative biting rate is drawn from a Log-normal distribution with a mean of 1:

$$log(\zeta_i) \sim N\left(\frac{-\sigma^2}{2}, \sigma^2\right). \quad (3)$$

The EIR $\varepsilon_i(a,t)$ and force of infection $\Lambda_i(a,t)$ experienced by individual *i* with age *a* at time *t* are given by

$$\varepsilon_i(a,t) = \varepsilon_0(t)\zeta_i\psi_i(a)$$
$$\Lambda_i(a,t) = b_i(t)\varepsilon_i(a,t). \quad (4)$$

Here $\varepsilon_0(t)$ is the mean EIR experienced by adults at time *t* and $b_i(t)$ is the probability that an infectious bite leads to a patent infection. The latter is determined by the level of pre-erythrocytic immunity (see below).

1.1.2 Naturally-acquired immunity

We capture the natural acquisition and loss of immunity dynamically through its relationship with both age and exposure. Newborns acquire a level of maternal immunity to clinical disease and severe disease at birth, denoted I_{CM} and I_{VM} respectively. The level at birth is set as a proportion, P_M, of the acquired immunity to clinical and severe malaria respectively of a randomly chosen 15–35-year-old in the population with the same heterogeneity level. This decays exponentially at a constant rate $r_M = 1/d_M$.

Acquired immunity to infection (pre-erythrocytic immunity) develops at older ages, is boosted by one level following each infected bite provided it is at least u_B days since the last exposure, and decays exponentially in between exposures with rate $r_B = \frac{1}{d_B}$. Blood stage immunity is assumed to control parasite density and hence affect the probability of developing severe disease, clinical disease and ultimately the detectability of asymptomatic infection. Acquired immunity to each of severe disease, clinical disease and detectability of infection is tracked separately, is boosted by one level following each patent infection provided it is at least u_V, u_C or $u_D$days respectively since the last exposure, and decays exponentially between exposures with rate $\frac{r_{VA}=1}{d_{VA}}$, $r_{CA} = \frac{1}{d_{CA}}$ and $r_{ID} = \frac{1}{d_{ID}}$ respectively.

All immunity levels are converted to individual time-dependent probabilities using Hill functions. The probability that individual *i* who is exposed to an infectious bite at time *t* develops a patent infection is given by:

$$b_i(t) = b_0\left(b_1 + \frac{1-b_1}{1+\left(\frac{I_B(i,t)}{I_{B0}}\right)^{\kappa_B}}\right) \quad (5)$$

where b_0 is the probability of infection with no immunity, $b_0 b_1$ is the minimum probability, I_{B0} and κ_B are scale and shape parameters respectively and $I_B(i,t)$ is the level of pre-erythrocytic immunity of individual *i* at time *t*.

The probability that individual *i* develops clinical disease at time *t* conditional on having been infected is given by:

$$\phi_i(t) = \phi_0\left(\phi_1 + \frac{1-\phi_1}{1+\left(\frac{(I_{CA}(i,t)+I_{CM}(i,t))}{I_{C0}}\right)^{\kappa_C}}\right) \quad (6)$$

where ϕ_0 is the probability of disease with no immunity, $\phi_0\phi_1$ is the minimum probability, I_{C0} and κ_C are scale and shape parameters respectively, $I_{CA}(i,t)$ is the level of acquired immunity to clinical disease and $I_{CM}(i,t)$ is the level of maternally acquired immunity to clinical disease of individual *i* at time *t*.

The probability that individual *i* develops severe disease at time *t* and age *a* conditional on being infected is defined as:

$$\theta_i(a,t) = \theta_0\left(\theta_1 + \frac{1-\theta_1}{1+f_V(i,a)\left(\frac{(I_{VA}(i,t)+I_{VM}(i,t))}{I_{V0}}\right)^{\kappa_V}}\right) \quad (7)$$

where θ_0 is the probability of disease with no immunity, $\theta_0\theta_1$ is the minimum probability, I_{V0} and κ_V are scale and shape parameters respectively, $I_{VA}(i,t)$ is the level of acquired immunity to severe disease, $I_{VM}(i,t)$ is the level of maternally acquired immunity to severe disease of individual *i* at time *t* and

$$f_V(i,a) = 1 - \frac{(1-f_{V0})}{\left(1+\left(\frac{a}{a_V}\right)^{\gamma_V}\right)} \quad (8)$$

is an age-dependent (physiological) modifier of the risk of severe disease, where f_{V0}, a_V and γ_V are parameters.

The detectability by microscopy of an asymptomatic infection in individual *i* of age *a* at time *t* is given by:

$$q_i(a,t) = d_1 + \frac{(1-d_{min}}{\left(\left(\frac{1+I_D(i,t)}{I_{D0}}\right)^{\kappa_D} f_D(i,a)\right)} \quad (9)$$

where d_{min} is the minimum probability of detection, I_{D0} and κ_D are scale and shape parameters respectively, $I_D(i,t)$ is the level of acquired immunity to the detectability of infection of individual *i* at time *t* and

$$f_D(i,a) = 1 - \frac{(1-f_{D0})}{\left(1+\left(\frac{a}{a_D}\right)^{\gamma_D}\right)} \quad (10)$$

is an age-dependent (physiological) modifier of the detectability of infection where f_{D0}, a_D and γ_D are parameters.

1.1.3 Onward infectivity to mosquitoes

Each infection state is assumed to be onwardly infectious to mosquitoes who bite an individual, with the highest infectivity associated with the states in which parasite density is highest (i.e. disease). Onwards infectiousness is $c_D$and c_U in states $D$and U respectively, and c_T following treatment. In state A infectiousness is modified by the detectability of individual i, q_i, and is given by the function as $c_U + (c_D + c_U)\, q_i{}^{\gamma_I}$.

1.1.4 Severe disease and mortality

We use the model estimates of clinical incidence to derive estimates of severe disease incidence and malaria-associated mortality. Following Griffin *et al* [3], incidence of severe malaria requiring hospitalisation in the age range a_L to a_U at time *t* is given by:

$$\lambda_H(t,(a_L,a_U)) = \frac{\sum_{i\,:\,a_L< a_i(t) <a_U}((1-f_T)+f_T f_{VT})\Lambda_i(t)\ \theta_i(t)}{\#\{i\,:\,a_L< a_i(t) <a_U\}} \quad (11)$$

where $\Lambda_i(t)$ is the force of infection experienced by individual *i* at time *t* and $\theta_i(t)$ the probability that individual *i* develops severe disease at time *t* upon being infected. Malaria-related mortality is assumed to be proportional to the incidence of severe disease due to malaria and is defined as:

$$\mu(t,(a_L,a_U)) = v\lambda_H(t,(a_L,a_U)) \quad (12)$$

where parameter v is a scaling factor. Individuals receiving treatment are assumed to experience a reduction, f_{VT}, in the probability of disease progression to severe disease and hence death.

1.1.5 Vector model

We model infection in the mosquito population using the deterministic model previously described by White *et al* but with an equivalent compartmental stochastic form for adult female mosquitoes [4]. Adult (female) mosquitoes are assumed to lay eggs at rate β. Upon hatching from eggs, larvae progress through early and late larvae stages (E and L compartments) before developing to the pupal stage (P_L). The larval stages are regulated by density dependent mortality, with a time-varying carrying-capacity, K, that represents the ability of the environment to sustain breeding sites through different periods of the year and with the density of larvae in relation to the carrying-capacity regulated by a parameter γ. The carrying-capacity determines the mosquito density and hence the baseline transmission intensity in the absence of interventions. The differential equations for the larval stages are given by:

$$\frac{dE}{dt} = \beta M - \mu_E \left(1 + \frac{E+L}{K}\right) E - \frac{E}{d_{EL}}$$
$$\frac{dL}{dt} = \frac{E}{d_{EL}} - \mu_L \left(1 + \gamma \frac{E+L}{K}\right) L - \frac{L}{d_L} \qquad (13)$$
$$\frac{dP_L}{dt} = \frac{L}{d_L} - \mu_P P_L - \frac{P_L}{d_{PL}}$$
$$\frac{dS_M}{dt} = \frac{P_L}{2d_{PL}} - \mu_M S_M$$

We assume that 50% of the emergent adult mosquitoes are female and all enter the susceptible state (S_M). The rate at which adult female mosquitoes become infected is a function of the infectiousness of the human population including an appropriate time-lag (t_l) to account for the period between humans becoming infected and becoming infectious. The force of infection experienced by mosquitoes (Λ_M) is given by:

$$\Lambda_M(t) = \frac{\alpha}{\omega} \int_0^\infty \int_0^\infty \zeta\psi(a)(c_D D(\zeta, a, t - t_l) + c_T T\,(\zeta, a, t - t_l) + c_A A(\zeta, a, t - t_l) + c_U U(\zeta, a, t - t_l) da d\zeta \quad (14)$$

where α is the biting rate on humans,

$$\alpha = Q_0/\delta, \qquad (15)$$

Q_0 quantifies the level of anthropophagy, and δ is the mean time between feeds. The parameter ω represents a normalising constant for the biting rate over all ages:

$$\omega = \int_0^{\infty\int} \psi(a)\, g(\,a)\, da \qquad (16)$$

where $g(a)$ is the human age distribution. There is a fixed delay τ_M before female mosquitoes become infectious to humans (I_M) and they are assumed to remain infectious after this.

1.1.6 Seasonality

Seasonality is incorporated through a time-varying carrying capacity

$$K(t) = K_0 \frac{R(t)}{\bar{R}} \qquad (17)$$

where K_0 is the mean carrying capacity, $\bar{R}$ the mean rainfall over the year and $R(t)$, the time varying seasonal curve. The latter is obtained from rainfall data using the first three frequencies of a Fourier transform of the daily rainfall data:

$$R(t) = g_0 + \sum_{i=1}^{3} g_i \cos(\,2\pi t i) + h_i \sin(\,2\pi t i), \qquad (18)$$

obtained from the US Climate Prediction Center for sub-Saharan Africa between 2002 and 2009 [5].

1.1.7 Vector bionomics

Within Africa the relative abundance of the three dominant vector species in each administrative unit (*An.gambiae s.s.*, *An. Arabiensis* and *An.funestus*) were based on spatial estimates made by the Malaria Atlas Project [6]. The characterising bionomics parameters for these African vector species are shown in Table B.

Table B: Vector bionomics parameters.

Bionomics trait	*An gambiae s.s**	*An arabiensis*	*An funestus*
Anthropophagy	0.92	0.71	0.94
Endophily	0.81	0.42	0.81
% bites indoors	0.97	0.96	0.98
% bites indoors and in bed	0.89	0.90	0.90
Daily mortality of adults with no interventions	0.132	0.132	0.112

*includes *An.coluzzi*

1.2 Parameter values

All baseline model parameter estimates are included in Table C. These are collated from previous publications. and are based on a number of model-fitting exercises and analyses of experimental data [4,7–9].

Table C: Model parameters.

Parameter	Symbol	Estimate	Reference
**Human infection duration (days)**			
Latent period	d_E	12	[10]
Patent infection	d_A	195	[10,11]
Clinical disease (treated)	d_T	5	[12]
Clinical disease (untreated)	d_D	5	[13]
Sub-patent infection	d_U	110	Fitted [9]
**Treatment**			

Probability of seeking treatment if clinically diseased	f_T	Varies by admin-1 unit	Estimated from Demographic and Health Surveys [14]
**Age and heterogeneity**			
Age-dependent biting parameter	ρ	0.85	[15,16]
Age-dependent biting parameter	a_0	8 years	[15,16]
Variance of the log heterogeneity in biting rates	σ^2	1.67	[17]
**Immunity reducing probability of infection**			
Maximum probability due to no immunity	b_0	0.590076	Fitted [7]
Maximum relative reduction due to immunity	b_1	0.5	[18]
Inverse of decay rate	d_B	10 years	[18]
Scale parameter	I_{B0}	43.8787	Fitted [7]
Shape parameter	κ_B	2.15506	Fitted [7]
Duration in which immunity is not boosted	u_B	7.19919 days	Fitted [7]
New-born immunity relative to mother's	P_{CM}	0.774368	Fitted [7]
**Immunity reducing probability of clinical disease**			
Maximum probability due to no immunity	ϕ_0	0.791666	Fitted [7]
Maximum relative reduction due to immunity	ϕ_1	0.000737	Fitted [7]
Inverse of decay rate	d_{CA}	30 years	[19]
Scale parameter	I_{C0}	18.02366	Fitted [7]
Shape parameter	κ_C	2.36949	Fitted [7]
Duration in which immunity is not boosted	u_C	6.06349 days	Fitted [7]
Inverse of decay rate of maternal immunity	d_M	67.6952 days	Fitted [7]
**Immunity reducing probability of detection**			
Minimum probability due to maximum immunity	d_1	0.161527	Fitted [7]
Inverse of decay rate	d_{ID}	10 years	[18]
Scale parameter	I_{D0}	1.577533	Fitted [7]
Shape parameter	κ_D	0.476614	Fitted [7]
Duration in which immunity is not boosted	u_D	9.44512 days	Fitted [7]
Scale parameter relating age to immunity	a_D	21.9 years	Fitted [7]
Timescale with which immunity changes with age	f_{D0}	0.007055	Fitted [7]
Shape parameter relating age to immunity	γ_D	4.8183	Fitted [7]
**Immunity reducing probability of severe disease and mortality**			
Maximum probability due to no immunity	θ_0	0.0749886	Fitted [8]
Maximum relative reduction due to immunity	θ_1	0.0001191	Fitted [8]
Scale parameter	I_{V0}	1.09629	Fitted [8]
Shape parameter	κ_V	2.00048	Fitted [8]
Inverse of decay rate	d_{VA}	30 years	[19]
Duration in which immunity is not boosted	u_V	11.4321 days	Fitted [8]
Inverse of decay rate of maternal immunity	d_{VM}	76.8365 days	Fitted [8]
New-born immunity relative to mother's	P_{VM}	0.195768	Fitted [8]
Reduced probability of death due to treatment	f_{VT}	0.5	[1]
Age-dependent severe disease risk modifier parameter	f_{V0}	0.141195	Fitted [8]
Age-dependent severe disease risk modifier parameter	a_V	2493.41	Fitted [8]
Age-dependent severe disease risk modifier parameter	γ_V	2.91282	Fitted [8]
Mortality scaling factor from severe disease	ν	0.215	[9]
**Infectiousness to mosquitoes**			
Lag from parasites to infectious gametocytes	d_g	12 days	[20]
Untreated disease	c_D	0.068	[21]
Treated disease	c_T	0.021896	[22]
Sub-patent infection	c_U	0.0062	Fitted [9]
Parameter for infectiousness of state *A*	γ_1	1.82425	Fitted [9]
**Adult mosquito population model**			
Daily mortality of adults with no interventions	μ_M	Varies by species – see above	
Mean time between feeds	δ	3 days	[23,24]
Extrinsic incubation period	d_{EM}	10 days	[25]
**Larval model**			
Average number of eggs laid per female mosquito per day	β	21.2/day	Fitted [4]
Early instar larval developmental period	d_E	6.64 days	Fitted [4]
Late instar developmental period	d_L	3.72 days	Fitted [4]
Pupal developmental period	d_P	0.643 days	Fitted [4]
Mortality rate of early-stage larvae (density dependent)	μ_E	0.0338/day	Fitted [4]
Mortality rate of late-stage larvae (density dependent)	μ_L	0.0348/day	Fitted [4]
Mortality rate of pupae (density independent)	μ_P	0.249/day	Fitted [4]
Effect of density dependence on late instars relative to early instars	γ	13.25	Fitted [4]

1.3 Malaria interventions

The model incorporates four current interventions – long-lasting insecticide treated nets (LLINs), indoor residual spraying (IRS), seasonal malaria chemoprevention (SMC) and treatment of uncomplicated malaria – using models developed previously. A full description of these models is provided elsewhere [1]. The vaccine model builds on earlier work [26,27], and is described briefly below.

1.3.1 Antibody model of vaccine efficacy

Following the approach taken in White *et al* [27], we simulate the RTS,S-induced anti-CSP antibody titres following the third vaccine dose as a biphasic model where the antibody titre at time t post-vaccination is given by:

$$CSP(t) = CSP_{\text{peak}}\left(\rho_{\text{peak}}e^{-r_s t} + (1-\rho_{\text{peak}})e^{-r_l t}\right) \qquad (19)$$

where CSP_{peak} is the peak anti-CSP antibody titre, ρ_{peak} is the proportion of antibody response that is short lived, and r_s and r_l are the rates of decay for the short lived and long lived components respectively. We use a similar process to model antibody levels following the fourth dose but allowing the peak titre to be lower. Therefore, for the fourth dose given at time t_{fourth} anti-CSP antibody titre is given by:

$$CSP(t) = CSP_{\text{fourth}}\left(\rho_{\text{fourth}}e^{-r_s(t-t_{\text{fourth}})} + (1-\rho_{\text{fourth}})e^{-r_l(t-t_{\text{fourth}})}\right). \qquad (20)$$

The vaccine efficacy over time is then obtained using a dose-response curve:

$$V(t) = V_{\max}\left(1 - \frac{1}{1+\left(\frac{CSP(t)}{\beta}\right)^{\alpha}}\right) \qquad (21)$$

where V_{max} is the maximum efficacy against infection and α and β are the estimated shape and scale parameters respectively. The vaccine parameters are summarised in Table D. Variation between individuals is captured by drawing the parameters noted from a Normal distribution on the log-scale.

Table D: RTS,S antibody model parameters, definitions and values. The model parameters are those reported in Table 3 of White et al. [27]. The values for the peak anti-CSP following the third and fourth dose were each calculated as the median of the 11 site values in the phase 3 RTS,S/AS01 trial [27].

Parameter	Description	Value
$1/r_s$	Half-life of short-lived antibodies (mean in days)	45
$1/r_l$	Half-life of long-lived antibodies (days, sampled from log-normal distribution)	591
CSP_{peak}	Peak anti-CSP following the third dose (geometric mean, EU/mL)	621
ρ_{peak}	Proportion of short-lived response following primary schedule	0.88
CSP_{fourth}	Peak anti-CSP following the fourth dose (mean on log-scale)	277
ρ_{fourth}	Proportion of short-lived response following the fourth dose	0.70
V_{max}	Maximum efficacy against infection	0.93
α	Dose-response shape parameter	0.74
β	Dose-response scale parameter (EU/mL)	99.2

2 References

1. Winskill P, Slater HC, Griffin JT, Ghani AC, Walker PGT. The US President's Malaria Initiative, Plasmodium falciparum transmission and mortality: A modelling study. PLoS Med. 2017;649: 1–14.
2. United Nations. United Nations World Population Prospects. 2015 [cited 18 Apr 2019]. Available: https://population.un.org/wpp/
3. Griffin JT, Bhatt S, Sinka ME, Gething PW, Lynch M, Patouillard E, et al. Potential for reduction of burden and local elimination of malaria by reducing Plasmodium falciparum malaria transmission: A mathematical modelling study. Lancet Infect Dis. 2016;16: 465–472. doi:10.1016/S1473-3099(15)00423-5
4. White MT, Griffin JT, Churcher TS, Ferguson NM, Basáñez MG, Ghani AC. Modelling the impact of vector control interventions on Anopheles gambiae population dynamics. Parasit Vectors. 2011;4: 153. doi:10.1186/1756-3305-4-153
5. National Weather Service. Climate Prediction Center: international desk data archive. [cited 2 Jun 2016]. Available: https://www.cpc.ncep.noaa.gov/products/international/africa/africa.shtml
6. Sinka ME, Golding N, Massey NC, Wiebe A, Huang Z, Hay SI, et al. Modelling the relative abundance of the primary African vectors of malaria before and after the implementation of indoor, insecticide-based vector control. Malar J. 2016;15: 1–10. doi:10.1186/s12936-016-1187-8
7. Griffin JT, Ferguson NM, Ghani AC. Estimates of the changing age-burden of Plasmodium falciparum malaria disease in sub-Saharan Africa. Nat Commun. 2014;5: 3136. doi:10.1038/ncomms4136
8. Griffin JT, Hollingsworth TD, Reyburn H, Drakeley CJ, Riley EM, Ghani AC. Gradual acquisition of immunity to severe malaria with increasing exposure. Proc R Soc B Biol Sci. 2015;282. doi:10.1098/rspb.2014.2657
9. Griffin JT, Bhatt S, Sinka ME, Gething PW, Lynch M, Patouillard E, et al. Potential for reduction of burden and local elimination of malaria by reducing Plasmodium falciparum malaria transmission: A mathematical modelling study. Lancet Infect Dis. 2016;16: 465–472. doi:10.1016/S1473-3099(15)00423-5
10. Eyles DE, Young MD. The duration of untreated or inadequately treated Plasmodium falciparum infections in the human host. J Natl Malar Soc. 1951;10: 327–336.
11. Falk N, Maire N, Sama W, Owusu-Agyei S, Smith T, Beck HP, et al. Comparison of PCR-RFLP and genescan-based genotyping for analyzing infection dynamics of Plasmodium falciparum. Am J Trop Med Hyg. 2006;74: 944–950. doi:10.4269/ajtmh.2006.74.944
12. Zwang J, Ashley EA, Karema C, D'Alessandro U, Smithuis F, Dorsey G, et al. Safety and efficacy of dihydroartemisinin-piperaquine in falciparum malaria: a prospective multi-centre individual patient data analysis. Diemert DJ, editor. PLoS One. 2009;4: e6358. doi:10.1371/journal.pone.0006358
13. Miller MJ. Observations on the natural history of malaria in the semi-resistant West African. Trans R Soc Trop Med Hyg. 1958;52: 152–168. doi:10.1016/0035-9203(58)90036-1
14. The DHS Program. Demographic and Health Surveys. 2019 [cited 29 Apr 2019]. Available: https://dhsprogram.com/
15. Carnevale P, Frézil JL, Bosseno MF, Le Pont F, Lancien J. [The aggressiveness of Anopheles gambiae A in relation to the age and sex of the human subjects]. Bull World Health Organ. 1978;56: 147—154. Available: https://europepmc.org/articles/PMC2395533
16. Port GR, Boreham PFL, Bryan JH. The relationship of host size to feeding by mosquitoes of the *Anopheles gambiae* Giles complex (Diptera: Culicidae). Bull Entomol Res. 1980;70: 133–144. doi:10.1017/S0007485300009834
17. Smith T, Charlwood JD, Takken W, Tanner M, Spiegelhalter DJ. Mapping the densities of malaria vectors within a single village. Acta Trop. 1995;59: 1–18. doi:10.1016/0001-706x(94)00082-c
18. Griffin JT, Hollingsworth TD, Okell LC, Churcher TS, White M, Hinsley W, et al. Reducing Plasmodium falciparum malaria transmission in Africa: A model-based evaluation of intervention strategies. PLoS Med. 2010;7. doi:10.1371/journal.pmed.1000324
19. Deloron P, Chougnet C. Is immunity to malaria really short-lived? Parasitol Today. 1992;8: 375–378. doi:10.1016/0169-4758(92)90174-Z
20. Ross A, Killeen G, Smith T. Relationships between host infectivity to mosquitoes and asexual parasite density in Plasmodium falciparum. Am J Trop Med Hyg Trop Med Hyg. 2006;75: 32–37. doi:10.4269/ajtmh.2006.75.32
21. Collins WE, Jeffery GM. A retrospective examination of mosquito infection on humans infected with Plasmodium falciparum. Am J Trop Med Hyg. 2003;68: 366–371.
22. Dunyo S, Milligan P, Edwards T, Sutherland C, Targett G, Pinder M. Gametocytaemia after drug treatment of asymptomatic Plasmodium falciparum. PLoS Clin Trials. 2006;1: e20. doi:10.1371/journal.pctr.0010020
23. Killeen GF, McKenzie FE, Foy BD, Schieffelin C, Billingsley PF, Beier JC. A simplified model for predicting malaria entomologic inoculation rates based on entomologic and parasitologic parameters relevant to control. Am J Trop Med Hyg. 2000;62: 535–544. doi:10.4269/ajtmh.2000.62.535
24. Mendis C, Jacobsen JL, Gamage-Mendis A, Bule E, Dgedge M, Thompson R, et al. Anopheles arabiensis and An. funestus are equally important vectors of malaria in Matola coastal suburb of Maputo, southern Mozambique. Med Vet Entomol. 2000;14: 171–180. doi:10.1046/j.1365-2915.2000.00228.x

25. Gu W, Mbogo CM, Githure JI, Regens JL, Killeen GF, Swalm CM, et al. Low recovery rates stabilize malaria endemicity in areas of low transmission in coastal Kenya. Acta Trop. 2003;86: 71–81. doi:10.1016/S0001-706X(03)00020-2
26. Penny MA, Verity R, Bever CA, Sauboin C, Galactionova K, Flasche S, et al. Public health impact and cost-effectiveness of the RTS,S/AS01 malaria vaccine: a systematic comparison of predictions from four mathematical models. Lancet. 2016;387: 367–375. doi:10.1016/S0140-6736(15)00725-4
27. White MT, Verity R, Griffin JT, Asante KP, Owusu-Agyei S, Greenwood B, et al. Immunogenicity of the RTS,S/AS01 malaria vaccine and implications for duration of vaccine efficacy: Secondary analysis of data from a phase 3 randomised controlled trial. Lancet Infect Dis. 2015;15: 1450–1458. doi:10.1016/S1473-3099(15)00239-X
