# Supplementary material for: Estimated impact of RTS,S/AS01 malaria vaccine allocation strategies in sub-Saharan Africa: A modelling study
Source: PLoS Med. 2020 Nov 30;17(11):e1003377. doi: 10.1371/journal.pmed.1003377 (PMC7703928; doi:10.1371/journal.pmed.1003377)
Supplement: S1 Table — ISO, International Organization for Standardization. (DOCX) [file pmed.1003377.s002.docx]

| **Country** | **Code** |
| --- | --- |
| Angola | AGO |
| Benin | BEN |
| Burkina Faso | BFA |
| Burundi | BDI |
| Cameroon | CMR |
| Central African Republic | CAF |
| Chad | TCD |
| Congo | COG |
| Côte d'Ivoire | CIV |
| Democratic Republic of the Congo | COD |
| Equatorial Guinea | GNQ |
| Gabon | GAB |
| Ghana | GHA |
| Guinea | GIN |
| Guinea-Bissau | GNB |
| Kenya | KEN |
| Liberia | LBR |
| Malawi | MWI |
| Mali | MLI |
| Mozambique | MOZ |
| Niger | NER |
| Nigeria | NGA |
| Sierra Leone | SLE |
| South Sudan | SSD |
| Tanzania | TZA |
| Togo | TGO |
| Uganda | UGA |
| Zambia | ZMB |
