# Supplementary material for: Estimated impact of RTS,S/AS01 malaria vaccine allocation strategies in sub-Saharan Africa: A modelling study
Source: PLoS Med. 2020 Nov 30;17(11):e1003377. doi: 10.1371/journal.pmed.1003377 (PMC7703928; doi:10.1371/journal.pmed.1003377)
Supplement: S2 Table — The impact is the annual events averted in 0- to 5-year-old children in the first 5 years following vaccine introduction, for the 4-dose schedule. 95% CrI represents the 95% credible interval, based on 50 parameter draws. The countries introducing in each scenario are listed in alphabetical order. Three-letter codes for the countries are available in S1 Table. (DOCX) [file pmed.1003377.s003.docx]

| Dose constraint (million) | Baseline intervention scenario | Vaccine coverage scenario | Clinical cases averted in thousands (95% CrI) | Severe cases averted in thousands (95% CrI) | Deaths averted in thousands (95% CrI) | Clinical cases averted per 1,000 doses | Countries introducing |
| --- | --- | --- | --- | --- | --- | --- | --- |
| 10 | Maintain 2016 | Realistic coverage | 1757 (1169–2699) | 49 (24–77) | 8 (4–13) | 180 | BEN, BFA, COG, GAB, GHA, GNQ, SLE, TGO |
| 10 | Maintain 2016 | 100% coverage | 2226 (1516–3353) | 58 (29–91) | 10 (5–16) | 225 | BFA, CAF, GAB, GHA, GIN, GNQ, SLE, TGO |
| 10 | High | Realistic coverage | 1560 (1043–2432) | 42 (21–68) | 5 (3–9) | 158 | BFA, CAF, GAB, GHA, GIN, GNQ, SLE, TGO |
| 10 | High | 100% coverage | 2058 (1398–3125) | 54 (27–87) | 7 (3–11) | 206 | CAF, GHA, GIN, MOZ, SLE |
| 20 | Maintain 2016 | Realistic coverage | 3002 (1972–4712) | 86 (42–140) | 14 (7–23) | 153 | BEN, BFA, CAF, COG, GAB, GHA, GIN, GNQ, LBR, MOZ, SLE, TGO, ZMB |
| 20 | Maintain 2016 | 100% coverage | 3946 (2677–6012) | 112 (56–175) | 19 (10–30) | 198 | BEN, BFA, CAF, COG, GHA, GIN, GNQ, LBR, MLI, MOZ, SLE, TGO |
| 20 | High | Realistic coverage | 2678 (1779–4294) | 79 (39–130) | 10 (5–17) | 135 | BFA, CAF, CIV, GHA, GIN, GNQ, MOZ, SLE, TGO, ZMB |
| 20 | High | 100% coverage | 3356 (2239–5245) | 99 (49–158) | 13 (6–20) | 168 | BEN, BFA, CAF, COG, GHA, GIN, GNQ, LBR, MLI, MOZ, SLE, TGO |
| 30 | Maintain 2016 | Realistic coverage | 4254 (2785–6788) | 128 (63–205) | 22 (11–35) | 143 | BEN, BFA, COD, GAB, GHA, GIN, GNQ, MOZ, SLE, TGO, ZMB |
| 30 | Maintain 2016 | 100% coverage | 5234 (3522–8209) | 157 (78–245) | 27 (14–43) | 182 | BEN, BFA, CAF, COD, COG, GAB, GHA, GIN, GNQ, LBR, MOZ, SLE, TGO |
| 30 | High | Realistic coverage | 3640 (2354–5924) | 113 (55–186) | 15 (7–24) | 122 | BEN, BFA, COD, GAB, GHA, GIN, GNQ, MOZ, SLE, TGO, ZMB |
| 30 | High | 100% coverage | 4639 (3046–7420) | 144 (71–229) | 19 (9–30) | 155 | BFA, CAF, COD, GAB, GHA, GIN, GNQ, LBR, MLI, MOZ, SLE, TGO |
| 40 | Maintain 2016 | Realistic coverage | 5125 (3314–8274) | 163 (79–261) | 28 (14–45) | 132 | BEN, BFA, CAF, COD, COG, GAB, GHA, GIN, GNQ, LBR, MOZ, MWI, NER, SLE, TGO, ZMB |
| 40 | Maintain 2016 | 100% coverage | 6638 (4413–10476) | 210 (104–326) | 37 (18–57) | 169 | BEN, BFA, CAF, COD, COG, GAB, GHA, GIN, GNQ, LBR, MLI, MOZ, MWI, NER, SLE, TGO |
| 40 | High | Realistic coverage | 4420 (2821–7347) | 145 (70–239) | 19 (9–31) | 111 | BEN, BFA, CAF, CIV, COD, GHA, GIN, GNQ, LBR, MLI, MOZ, MWI, SLE, TGO, ZMB |
| 40 | High | 100% coverage | 5645 (3658–9226) | 184 (90–297) | 24 (12–38) | 141 | BEN, BFA, CAF, CIV, COD, GHA, GIN, GNQ, LBR, MLI, MOZ, MWI, SLE, TGO, ZMB |
| 50 | Maintain 2016 | Realistic coverage | 5924 (3801–9623) | 193 (94–309) | 33 (16–54) | 122 | BEN, BFA, CAF, CIV, CMR, COD, COG, GAB, GHA, GIN, GNQ, LBR, MLI, MOZ, MWI, NER, SLE, TGO, ZMB |
| 50 | Maintain 2016 | 100% coverage | 8607 (5818–13382) | 254 (127–406) | 43 (21–68) | 173 | BEN, BFA, CAF, COG, GAB, GHA, GIN, GNQ, LBR, MLI, MOZ, NGA, SLE, TGO |
| 50 | High | Realistic coverage | 4947 (3096–8376) | 168 (79–280) | 22 (10–36) | 101 | BDI, BEN, BFA, CAF, CIV, COD, COG, GAB, GHA, GIN, GNQ, LBR, MLI, MOZ, MWI, SLE, TGO, UGA, ZMB |
| 50 | High | 100% coverage | 6281 (4010–10459) | 211 (103–346) | 27 (13–45) | 128 | BDI, BEN, BFA, CAF, CIV, COD, COG, GAB, GHA, GIN, GNQ, LBR, MLI, MOZ, MWI, SLE, TGO, UGA, ZMB |
| 60 | Maintain 2016 | Realistic coverage | 6550 (4155–10799) | 220 (106–356) | 38 (18–61) | 111 | BDI, BEN, BFA, CAF, CIV, CMR, COD, COG, GAB, GHA, GIN, GNQ, LBR, MLI, MOZ, MWI, NER, SLE, TCD, TGO, UGA, ZMB |
| 60 | Maintain 2016 | 100% coverage | 9982 (6712–15747) | 303 (150–483) | 51 (25–82) | 167 | BEN, BFA, CAF, COD, COG, GAB, GHA, GIN, GNQ, LBR, MOZ, NGA, SLE, SSD, TGO |
| 60 | High | Realistic coverage | 5282 (3250–9052) | 185 (86–312) | 24 (11–40) | 89 | BDI, BEN, BFA, CAF, CIV, CMR, COD, COG, GAB, GHA, GIN, GNQ, LBR, MLI, MOZ, MWI, NER, SLE, TCD, TGO, UGA, ZMB |
| 60 | High | 100% coverage | 6733 (4234–11347) | 235 (112–390) | 30 (15–50) | 114 | BDI, BEN, BFA, CAF, CIV, CMR, COD, COG, GAB, GHA, GIN, GNQ, LBR, MLI, MOZ, MWI, NER, SLE, TCD, TGO, UGA, ZMB |
