# Supplementary material for: Estimated impact of RTS,S/AS01 malaria vaccine allocation strategies in sub-Saharan Africa: A modelling study
Source: PLoS Med. 2020 Nov 30;17(11):e1003377. doi: 10.1371/journal.pmed.1003377 (PMC7703928; doi:10.1371/journal.pmed.1003377)
Supplement: S3 Table — The impact is the annual events averted in 0- to 5-year-old children in the first 5 years following vaccine introduction, for the 4-dose schedule. 95% CrI represents the 95% credible interval, based on 50 parameter draws. The countries introducing in each scenario are listed in alphabetical order. Three-letter codes for the countries are available in S1 Table. (DOCX) [file pmed.1003377.s004.docx]

| Dose constraint (million) | Baseline intervention scenario | Vaccine coverage scenario | Clinical cases averted in thousands (95% CrI) | Severe cases averted in thousands (95% CrI) | Deaths averted in thousands (95% CrI) | Clinical cases averted per 1,000 doses | Countries introducing |
| --- | --- | --- | --- | --- | --- | --- | --- |
| 10 | Maintain 2016 | Realistic coverage | 2461 (1658–3582) | 58 (28–90) | 10 (5–15) | 246 | BEN, BFA, CAF, CIV, CMR, COD, GHA, MOZ, MWI, NGA, SLE, TCD, TGO, UGA, ZMB |
| 10 | Maintain 2016 | 100% coverage | 3543 (2504–5025) | 72 (36–121) | 12 (6–20) | 354 | BFA, CAF, CIV, COD, GHA, GIN, GNQ, MOZ, MWI, NGA, TGO, ZMB |
| 10 | High | Realistic coverage | 2194 (1549–3290) | 54 (27–84) | 7 (4–11) | 219 | BFA, CAF, CIV, COD, GHA, GIN, LBR, MOZ, MWI, SLE, TCD, TGO, UGA, ZMB |
| 10 | High | 100% coverage | 2972 (2082–4251) | 66 (34–105) | 9 (4–13) | 297 | BFA, CAF, CIV, COD, GHA, GIN, MOZ, MWI, NGA, SLE, TCD, UGA, ZMB |
| 20 | Maintain 2016 | Realistic coverage | 4091 (2736–6166) | 102 (50–161) | 17 (9–27) | 205 | BEN, BFA, CAF, CIV, CMR, COD, COG, GHA, GIN, KEN, LBR, MLI, MOZ, MWI, NER, NGA, SLE, TCD, TGO, UGA, ZMB |
| 20 | Maintain 2016 | 100% coverage | 5962 (4208–8652) | 138 (70–219) | 23 (12–37) | 298 | BEN, BFA, CAF, CIV, CMR, COD, GHA, GIN, GNQ, KEN, MLI, MOZ, MWI, NER, NGA, SLE, TCD, TGO, UGA, ZMB |
| 20 | High | Realistic coverage | 3534 (2377–5424) | 95 (47–149) | 12 (6–19) | 177 | BEN, BFA, CAF, CIV, COD, GHA, GIN, KEN, LBR, MLI, MOZ, MWI, NER, NGA, SLE, TCD, TGO, UGA, ZMB |
| 20 | High | 100% coverage | 4924 (3407–7331) | 125 (64–193) | 16 (8–25) | 246 | BFA, CAF, CIV, COD, GHA, GIN, LBR, MLI, MOZ, MWI, NGA, SLE, TCD, TGO, UGA, ZMB |
| 30 | Maintain 2016 | Realistic coverage | 5328 (3502–8162) | 143 (70–225) | 24 (12–38) | 178 | BEN, BFA, CAF, CIV, CMR, COD, COG, GHA, GIN, KEN, LBR, MLI, MOZ, MWI, NER, NGA, SLE, TCD, TGO, UGA, ZMB |
| 30 | Maintain 2016 | 100% coverage | 7958 (5589–11819) | 199 (102–310) | 34 (17–52) | 265 | BEN, BFA, CAF, CIV, CMR, COD, COG, GHA, GIN, GNQ, KEN, LBR, MLI, MOZ, MWI, NER, NGA, SLE, TCD, TGO, UGA, ZMB |
| 30 | High | Realistic coverage | 4512 (2962–7164) | 131 (65–208) | 17 (8–27) | 150 | BDI, BEN, BFA, CAF, CIV, CMR, COD, GHA, GIN, KEN, LBR, MLI, MOZ, MWI, NER, NGA, SLE, TCD, TGO, UGA, ZMB |
| 30 | High | 100% coverage | 6411 (4348–9779) | 178 (90–274) | 23 (12–35) | 214 | BEN, BFA, CAF, CIV, COD, GHA, GIN, GNQ, KEN, LBR, MLI, MOZ, MWI, NER, NGA, SLE, TCD, TGO, UGA, ZMB |
| 40 | Maintain 2016 | Realistic coverage | 6350 (4111–9800) | 179 (87–281) | 31 (15–48) | 159 | BDI, BEN, BFA, CAF, CIV, CMR, COD, COG, GHA, GIN, GNQ, KEN, LBR, MLI, MOZ, MWI, NER, NGA, SLE, TCD, TGO, UGA, ZMB |
| 40 | Maintain 2016 | 100% coverage | 9542 (6618–14397) | 256 (131–395) | 43 (22–67) | 239 | BEN, BFA, CAF, CIV, CMR, COD, COG, GHA, GIN, GNQ, KEN, LBR, MLI, MOZ, MWI, NER, NGA, SLE, TCD, TGO, UGA, ZMB |
| 40 | High | Realistic coverage | 5210 (3323–8393) | 161 (78–255) | 21 (10–33) | 130 | BDI, BEN, BFA, CAF, CIV, CMR, COD, COG, GHA, GIN, KEN, LBR, MLI, MOZ, MWI, NER, NGA, SLE, TCD, TGO, UGA, ZMB |
| 40 | High | 100% coverage | 7556 (5035–11776) | 225 (113–348) | 29 (15–45) | 189 | BEN, BFA, CAF, CIV, CMR, COD, GHA, GIN, GNQ, KEN, LBR, MLI, MOZ, MWI, NER, NGA, SLE, TCD, TGO, UGA, ZMB |
| 50 | Maintain 2016 | Realistic coverage | 7160 (4582–11213) | 211 (101–331) | 36 (17–56) | 143 | BDI, BEN, BFA, CAF, CIV, CMR, COD, COG, GAB, GHA, GIN, GNQ, KEN, LBR, MLI, MOZ, MWI, NER, NGA, SLE, TCD, TGO, TZA, UGA, ZMB |
| 50 | Maintain 2016 | 100% coverage | 10789 (7380–16519) | 306 (155–473) | 52 (26–80) | 216 | AGO, BDI, BEN, BFA, CAF, CIV, CMR, COD, COG, GHA, GIN, GNQ, KEN, LBR, MLI, MOZ, MWI, NER, NGA, SLE, SSD, TCD, TGO, UGA, ZMB |
| 50 | High | Realistic coverage | 5717 (3561–9389) | 184 (87–296) | 24 (11–38) | 114 | BDI, BEN, BFA, CAF, CIV, CMR, COD, COG, GHA, GIN, KEN, LBR, MLI, MOZ, MWI, NER, NGA, SLE, TCD, TGO, UGA, ZMB |
| 50 | High | 100% coverage | 8383 (5489–13330) | 265 (132–413) | 34 (17–53) | 168 | BDI, BEN, BFA, CAF, CIV, CMR, COD, COG, GHA, GIN, GNQ, KEN, LBR, MLI, MOZ, MWI, NER, NGA, SLE, TCD, TGO, UGA, ZMB |
| 60 | Maintain 2016 | Realistic coverage | 7788 (4917–12373) | 240 (114–380) | 41 (19–64) | 130 | BDI, BEN, BFA, CAF, CIV, CMR, COD, COG, GAB, GHA, GIN, GNQ, KEN, LBR, MLI, MOZ, MWI, NER, NGA, SLE, TCD, TGO, TZA, UGA, ZMB |
| 60 | Maintain 2016 | 100% coverage | 11786 (7976–18295) | 351 (177–544) | 59 (30–92) | 196 | AGO, BDI, BEN, BFA, CAF, CIV, CMR, COD, COG, GAB, GHA, GIN, GNQ, KEN, LBR, MLI, MOZ, MWI, NER, NGA, SLE, SSD, TCD, TGO, TZA, UGA, ZMB |
| 60 | High | Realistic coverage | 6092 (3716–10184) | 204 (95–334) | 26 (12–43) | 102 | BDI, BEN, BFA, CAF, CIV, CMR, COD, COG, GHA, GIN, GNQ, KEN, LBR, MLI, MOZ, MWI, NER, NGA, SLE, TCD, TGO, UGA, ZMB |
| 60 | High | 100% coverage | 8958 (5765–14523) | 296 (146–468) | 38 (19–60) | 149 | BDI, BEN, BFA, CAF, CIV, CMR, COD, COG, GHA, GIN, GNQ, KEN, LBR, MLI, MOZ, MWI, NER, NGA, SLE, SSD, TCD, TGO, UGA, ZMB |
