# Supplementary material for: Estimated impact of RTS,S/AS01 malaria vaccine allocation strategies in sub-Saharan Africa: A modelling study
Source: PLoS Med. 2020 Nov 30;17(11):e1003377. doi: 10.1371/journal.pmed.1003377 (PMC7703928; doi:10.1371/journal.pmed.1003377)
Supplement: S4 Table — The impact is the annual events averted in 0- to 5-year-old children in the first 5 years following vaccine introduction, for the 4-dose schedule. The relative impact is the clinical cases averted per 1,000 doses relative to that for the corresponding scenarios without pilot site prioritisation. 95% CrI represents the 95% credible interval, based on 50 parameter draws. Note that the total doses required at the lowest dose constraint (10 million) was 10.3 million, in order to prioritise all 3 pilot countries. The countries introducing in each scenario are listed in alphabetical order. Three-letter codes for the countries are available in S1 Table. (DOCX) [file pmed.1003377.s005.docx]

| Dose constraint (million) | Baseline intervention scenario | Vaccine coverage scenario | Clinical cases averted in thousands (95% CrI) | Severe cases averted in thousands (95% CrI) | Deaths averted in thousands (95% CrI) | Clinical cases averted per 1,000 doses | Countries introducing | Relative impact |
| --- | --- | --- | --- | --- | --- | --- | --- | --- |
| 10.3 | Maintain 2016 | Realistic coverage | 1086 (694–1777) | 34 (16–58) | 6 (3–10) | 105 | GHA, MWI, KEN | 58% |
| 10.3 | Maintain 2016 | 100% coverage | 1204 (772–1963) | 39 (18–65) | 6 (3–11) | 116 | GHA, MWI, KEN | 52% |
| 10.3 | High | Realistic coverage | 918 (594–1557) | 29 (14–52) | 4 (2–7) | 89 | GHA, MWI, KEN | 56% |
| 10.3 | High | 100% coverage | 1009 (650–1708) | 33 (16–58) | 4 (2–7) | 97 | GHA, MWI, KEN | 47% |
| 20 | Maintain 2016 | Realistic coverage | 2653 (1730–4186) | 79 (38–130) | 13 (6–22) | 133 | SLE, TGO, BFA, MOZ, COG, GNQ, GAB, GHA, MWI, KEN | 87% |
| 20 | Maintain 2016 | 100% coverage | 3169 (2097–4949) | 93 (45–152) | 16 (8–26) | 159 | GIN, GNQ, TGO, SLE, MOZ, CAF, COG, LBR, GAB, GHA, MWI, KEN | 80% |
| 20 | High | Realistic coverage | 2270 (1495–3668) | 69 (33–116) | 9 (4–15) | 114 | SLE, BFA, TGO, MOZ, CAF, GNQ, GAB, GHA, MWI, KEN | 84% |
| 20 | High | 100% coverage | 2861 (1896–4509) | 84 (41–139) | 11 (5–18) | 144 | GIN, SLE, MOZ, BFA, GNQ, GHA, MWI, KEN | 86% |
| 30 | Maintain 2016 | Realistic coverage | 3701 (2381–5941) | 116 (56–190) | 19 (9–32) | 124 | SLE, TGO, BFA, MOZ, BEN, ZMB, GIN, NER, GNQ, GAB, GHA, MWI, KEN | 87% |
| 30 | Maintain 2016 | 100% coverage | 4744 (3163–7427) | 144 (71–230) | 25 (12–40) | 159 | GIN, GNQ, TGO, SLE, MOZ, BFA, CAF, COG, BEN, MLI, CIV, GHA, MWI, KEN | 87% |
| 30 | High | Realistic coverage | 3132 (2013–5202) | 100 (48–170) | 13 (6–22) | 104 | SLE, BFA, TGO, MOZ, ZMB, GIN, CIV, BEN, CAF, LBR, COG, GHA, MWI, KEN | 85% |
| 30 | High | 100% coverage | 4002 (2622–6455) | 126 (61–206) | 16 (8–27) | 134 | GIN, SLE, MOZ, BFA, TGO, CAF, MLI, ZMB, BEN, GNQ, LBR, GAB, GHA, MWI, KEN | 86% |
| 40 | Maintain 2016 | Realistic coverage | 4796 (3095–7781) | 151 (73–245) | 26 (12–42) | 123 | SLE, TGO, BFA, MOZ, BEN, COD, ZMB, GIN, COG, LBR, CAF, GNQ, GAB, GHA, MWI, KEN | 93% |
| 40 | Maintain 2016 | 100% coverage | 6211 (4136–9851) | 195 (96–307) | 34 (17–53) | 157 | GIN, GNQ, TGO, SLE, MOZ, BFA, CAF, COG, COD, BEN, MLI, LBR, GAB, GHA, MWI, KEN | 93% |
| 40 | High | Realistic coverage | 4129 (2627–6914) | 135 (64–227) | 17 (8–29) | 103 | SLE, BFA, TGO, MOZ, ZMB, GIN, COD, CIV, CAF, LBR, GNQ, GHA, MWI, KEN | 93% |
| 40 | High | 100% coverage | 5268 (3418–8605) | 170 (83–277) | 22 (11–36) | 132 | GIN, SLE, MOZ, BFA, TGO, CAF, COD, MLI, ZMB, GNQ, GAB, GHA, MWI, KEN | 94% |
| 50 | Maintain 2016 | Realistic coverage | 5854 (3749–9526) | 190 (92–307) | 33 (16–53) | 117 | SLE, TGO, BFA, MOZ, BEN, COD, ZMB, GIN, NER, COG, CIV, MLI, LBR, CAF, GNQ, GHA, MWI, KEN | 96% |
| 50 | Maintain 2016 | 100% coverage | 7488 (4942–11969) | 242 (119–383) | 42 (21–66) | 150 | GIN, GNQ, TGO, SLE, MOZ, BFA, CAF, COG, COD, BEN, MLI, NER, CIV, ZMB, LBR, GHA, MWI, KEN | 87% |
| 50 | High | Realistic coverage | 4764 (2993–8042) | 162 (77–272) | 21 (10–35) | 95 | SLE, BFA, TGO, MOZ, ZMB, GIN, COD, CIV, MLI, BEN, CAF, LBR, NER, COG, GNQ, GHA, MWI, KEN | 94% |
| 50 | High | 100% coverage | 6090 (3890–10099) | 206 (100–338) | 27 (13–44) | 122 | GIN, SLE, MOZ, BFA, TGO, CAF, COD, MLI, ZMB, CIV, BEN, GNQ, LBR, NER, COG, GHA, MWI, KEN | 95% |
| 60 | Maintain 2016 | Realistic coverage | 6551 (4153–10806) | 219 (105–357) | 38 (18–61) | 109 | SLE, TGO, BFA, MOZ, BEN, COD, ZMB, GIN, NER, COG, CIV, MLI, UGA, LBR, CAF, CMR, GNQ, GAB, GHA, MWI, KEN | 98% |
| 60 | Maintain 2016 | 100% coverage | 9405 (6304–14797) | 287 (142–461) | 48 (24–77) | 158 | GIN, GNQ, TGO, SLE, MOZ, BFA, NGA, CAF, COG, BEN, MLI, CIV, GAB, GHA, MWI, KEN | 95% |
| 60 | High | Realistic coverage | 5281 (3259–9058) | 184 (86–312) | 24 (11–40) | 88 | SLE, BFA, TGO, MOZ, ZMB, GIN, COD, CIV, MLI, BEN, UGA, CAF, LBR, NER, BDI, COG, GNQ, SSD, GHA, MWI, KEN | 99% |
| 60 | High | 100% coverage | 6733 (4242–11357) | 234 (112–390) | 30 (14–50) | 112 | GIN, SLE, MOZ, BFA, TGO, CAF, COD, MLI, ZMB, CIV, BEN, GNQ, UGA, LBR, NER, COG, BDI, SSD, GHA, MWI, KEN | 98% |
