# Supplementary material for: Estimated impact of RTS,S/AS01 malaria vaccine allocation strategies in sub-Saharan Africa: A modelling study
Source: PLoS Med. 2020 Nov 30;17(11):e1003377. doi: 10.1371/journal.pmed.1003377 (PMC7703928; doi:10.1371/journal.pmed.1003377)
Supplement: S5 Table — The impact is the annual events averted in 0- to 5-year-old children in the first 5 years following vaccine introduction, for the 4-dose schedule. The relative impact is the clinical cases averted per 1,000 doses relative to that for the corresponding scenarios without pilot site prioritisation. 95% CrI represents the 95% credible interval, based on 50 parameter draws. Note that the total doses required at the lowest dose constraint (10 million) was 10.3 million, in order to prioritise all 3 pilot countries. The countries introducing in each scenario are listed in alphabetical order. Three-letter codes for the countries are available in S1 Table. (DOCX) [file pmed.1003377.s006.docx]

| Dose constraint (million) | Baseline intervention scenario | Vaccine coverage scenario | Clinical cases averted in thousands (95% CrI) | Severe cases averted in thousands (95% CrI) | Deaths averted in thousands (95% CrI) | Clinical cases averted per 1,000 doses | Countries introducing | Relative impact |
| --- | --- | --- | --- | --- | --- | --- | --- | --- |
| 10.3 | Maintain 2016 | Realistic coverage | 1086 (694–1777) | 34 (16–58) | 6 (3–10) | 105 | GHA, MWI, KEN | 43% |
| 10.3 | Maintain 2016 | 100% coverage | 1204 (772–1963) | 39 (18–65) | 6 (3–11) | 116 | GHA, KEN, MWI | 33% |
| 10.3 | High | Realistic coverage | 918 (594–1557) | 29 (14–52) | 4 (2–7) | 89 | GHA, KEN, MWI | 41% |
| 10.3 | High | 100% coverage | 1009 (650–1708) | 33 (16–58) | 4 (2–7) | 97 | GHA, KEN, MWI | 33% |
| 20 | Maintain 2016 | Realistic coverage | 3220 (2113–4905) | 86 (41–139) | 14 (7–23) | 161 | BFA, MOZ, ZMB, SLE, TGO, CIV, COD, CMR, BEN, NGA, UGA, CAF, GHA, MWI, KEN | 79% |
| 20 | Maintain 2016 | 100% coverage | 4516 (3117–6667) | 108 (52–181) | 18 (9–30) | 226 | NGA, GIN, MOZ, BFA, CAF, ZMB, TGO, COD, CIV, SLE, GNQ, TCD, GHA, KEN, MWI | 76% |
| 20 | High | Realistic coverage | 2803 (1897–4430) | 79 (39–129) | 10 (5–17) | 140 | MOZ, BFA, ZMB, TGO, SLE, UGA, COD, CIV, GIN, LBR, BEN, CAF, TCD, GHA, KEN, MWI | 79% |
| 20 | High | 100% coverage | 3731 (2529–5622) | 96 (48–157) | 12 (6–20) | 187 | GIN, MOZ, BFA, NGA, ZMB, CAF, SLE, CIV, COD, TGO, UGA, TCD, GHA, KEN, MWI | 76% |
| 30 | Maintain 2016 | Realistic coverage | 4784 (3147–7405) | 130 (63–209) | 22 (11–35) | 159 | BFA, MOZ, ZMB, SLE, TGO, CIV, COD, CMR, BEN, NGA, UGA, NER, GIN, CAF, MLI, LBR, COG, TCD, GHA, MWI, KEN | 89% |
| 30 | Maintain 2016 | 100% coverage | 6871 (4778–10254) | 173 (87–277) | 29 (15–47) | 229 | NGA, GIN, MOZ, BFA, CAF, ZMB, TGO, COD, CIV, SLE, GNQ, MLI, CMR, NER, BEN, UGA, COG, TCD, GHA, KEN, MWI | 86% |
| 30 | High | Realistic coverage | 4074 (2672–6468) | 118 (57–190) | 15 (7–25) | 136 | MOZ, BFA, ZMB, TGO, SLE, UGA, COD, CIV, GIN, LBR, BEN, MLI, NGA, NER, CAF, TCD, GHA, KEN, MWI | 91% |
| 30 | High | 100% coverage | 5612 (3804–8620) | 154 (77–245) | 20 (10–32) | 187 | GIN, MOZ, BFA, NGA, ZMB, CAF, SLE, CIV, COD, TGO, UGA, MLI, BEN, LBR, TCD, GHA, KEN, MWI | 87% |
| 40 | Maintain 2016 | Realistic coverage | 5944 (3843–9220) | 167 (80–266) | 28 (14–45) | 149 | BFA, MOZ, ZMB, SLE, TGO, CIV, COD, CMR, BEN, NGA, UGA, NER, GIN, CAF, MLI, LBR, COG, GHA, MWI, KEN | 94% |
| 40 | Maintain 2016 | 100% coverage | 8799 (6099–13307) | 234 (118–368) | 39 (20–62) | 220 | NGA, GIN, MOZ, BFA, CAF, ZMB, TGO, COD, CIV, SLE, GNQ, MLI, CMR, NER, BEN, UGA, COG, LBR, TCD, GHA, KEN, MWI | 92% |
| 40 | High | Realistic coverage | 4946 (3167–7999) | 151 (72–244) | 19 (9–31) | 124 | MOZ, BFA, ZMB, TGO, SLE, UGA, COD, CIV, GIN, LBR, BEN, MLI, NGA, NER, CAF, CMR, BDI, TCD, GHA, KEN, MWI | 95% |
| 40 | High | 100% coverage | 7018 (4670–10930) | 206 (103–324) | 27 (13–42) | 175 | GIN, MOZ, BFA, NGA, ZMB, CAF, SLE, CIV, COD, TGO, UGA, MLI, BEN, LBR, NER, CMR, GNQ, TCD, GHA, KEN, MWI | 93% |
| 50 | Maintain 2016 | Realistic coverage | 6881 (4401–10786) | 202 (97–321) | 34 (16–55) | 138 | BFA, MOZ, ZMB, SLE, TGO, CIV, COD, CMR, BEN, NGA, UGA, NER, GIN, CAF, MLI, LBR, COG, BDI, TZA, GHA, MWI, KEN | 97% |
| 50 | Maintain 2016 | 100% coverage | 10294 (7062–15763) | 289 (146–450) | 49 (25–76) | 206 | NGA, GIN, MOZ, BFA, CAF, ZMB, TGO, COD, CIV, SLE, GNQ, MLI, CMR, NER, BEN, UGA, COG, LBR, SSD, TCD, GHA, KEN, MWI | 95% |
| 50 | High | Realistic coverage | 5551 (3467–9115) | 177 (84–288) | 23 (11–37) | 111 | MOZ, BFA, ZMB, TGO, SLE, UGA, COD, CIV, GIN, LBR, BEN, MLI, NGA, NER, CAF, CMR, BDI, COG, TCD, GHA, KEN, MWI | 97% |
| 50 | High | 100% coverage | 8078 (5303–12836) | 251 (125–395) | 32 (16–51) | 162 | GIN, MOZ, BFA, NGA, ZMB, CAF, SLE, CIV, COD, TGO, UGA, MLI, BEN, LBR, NER, CMR, GNQ, BDI, COG, TCD, GHA, KEN, MWI | 96% |
| 60 | Maintain 2016 | Realistic coverage | 7594 (4803–12058) | 231 (110–368) | 39 (19–62) | 127 | BFA, MOZ, ZMB, SLE, TGO, CIV, COD, CMR, BEN, NGA, UGA, NER, GIN, CAF, MLI, LBR, COG, BDI, TZA, GNQ, TCD, GHA, MWI, KEN | 98% |
| 60 | Maintain 2016 | 100% coverage | 11434 (7754–17739) | 337 (169–526) | 57 (28–89) | 191 | NGA, GIN, MOZ, BFA, CAF, ZMB, TGO, COD, CIV, SLE, GNQ, MLI, CMR, NER, BEN, UGA, COG, LBR, SSD, BDI, AGO, GAB, TCD, GHA, KEN, MWI | 97% |
| 60 | High | Realistic coverage | 5990 (3670–10004) | 199 (93–326) | 26 (12–42) | 100 | MOZ, BFA, ZMB, TGO, SLE, UGA, COD, CIV, GIN, LBR, BEN, MLI, NGA, NER, CAF, CMR, BDI, COG, GNQ, TCD, GHA, KEN, MWI | 98% |
| 60 | High | 100% coverage | 8771 (5669–14196) | 286 (141–455) | 37 (18–59) | 146 | GIN, MOZ, BFA, NGA, ZMB, CAF, SLE, CIV, COD, TGO, UGA, MLI, BEN, LBR, NER, CMR, GNQ, BDI, COG, SSD, TCD, GHA, KEN, MWI | 98% |
