# Supplementary material for: Estimated impact of RTS,S/AS01 malaria vaccine allocation strategies in sub-Saharan Africa: A modelling study
Source: PLoS Med. 2020 Nov 30;17(11):e1003377. doi: 10.1371/journal.pmed.1003377 (PMC7703928; doi:10.1371/journal.pmed.1003377)
Supplement: S6 Table — The impact is the annual events averted in 0- to 5-year-old children in the first 5 years following vaccine introduction. Ranking was performed at the country level for a dose constraint of 30 million doses available per year. Results are shown for the baseline intervention scenario “Maintain 2016 coverage.” 95% CrI represents the 95% credible interval, based on 50 parameter draws. The countries introducing in each scenario are listed in alphabetical order. Three-letter codes for the countries are available in S1 Table. (DOCX) [file pmed.1003377.s007.docx]

| Dose schedule | Vaccine coverage | Clinical cases averted in thousands (95% CrI) | Severe cases averted in thousands (95% CrI) | Deaths averted in thousands (95% CrI) | Clinical cases averted per 1,000 doses | Countries introducing: 3 doses | Countries introducing: 4 doses |
| --- | --- | --- | --- | --- | --- | --- | --- |
| 4 doses | 100% coverage | 5234 (3522–8209) | 157 (78–245) | 27 (14–43) | 182 | NA | BEN, BFA, CAF, COD, COG, GAB, GHA, GIN, GNQ, LBR, MOZ, SLE, TGO |
| 4 doses | D1–3 DTP3, D4 100% | 4435 (2904–7047) | 136 (66–213) | 24 (11–37) | 148 | NA | BEN, BFA, COD, COG, GHA, GIN, MOZ, MWI, SLE, TGO |
| 4 doses | Realistic coverage: D1–3 DTP3, D4 80% | 4254 (2785–6788) | 128 (63–205) | 22 (11–35) | 143 | NA | BEN, BFA, COD, GAB, GHA, GIN, GNQ, MOZ, SLE, TGO, ZMB |
| 4 doses | D1–3 DTP3, D4 60% | 4084 (2674–6554) | 124 (60–199) | 21 (10–34) | 137 | NA | BEN, BFA, COD, GAB, GHA, GIN, GNQ, MOZ, SLE, TGO, ZMB |
| Either schedule | 100% coverage | 5353 (3531–8374) | 165 (81–261) | 29 (14–45) | 179 | BEN, BFA, COD, COG, GIN, GNQ, MLI, NAM, SLE, TGO | CAF, GHA, MOZ, MWI |
| Either schedule | D1–3 DTP3, D4 100% | 4368 (2820–6973) | 135 (65–213) | 23 (11–37) | 146 | BFA, CAF, COG, GIN, GNQ, TGO | BEN, COD, GHA, LBR, MOZ, MWI, SLE |
| Either schedule | Realistic coverage: D1–3 DTP3, D4 80% | 4136 (2636–6682) | 130 (61–211) | 22 (10–36) | 141 | BEN, BFA, COD, COG, GAB, GHA, GIN, GNQ, LBR, MWI, NAM, TGO, ZMB | CAF, MOZ, SLE |
| Either schedule | D1–3 DTP3, D4 60% | 4179 (2661–6798) | 133 (62–216) | 23 (11–37) | 139 | BEN, BFA, COD, COG, GHA, GIN, GNQ, MLI, MOZ, MWI, SLE, TGO, ZMB | NA |
