# Supplementary figures and images for: Estimated impact of RTS,S/AS01 malaria vaccine allocation strategies in sub-Saharan Africa: A modelling study
Source: PLoS Med. 2020 Nov 30;17(11):e1003377. doi: 10.1371/journal.pmed.1003377 (PMC7703928; doi:10.1371/journal.pmed.1003377)

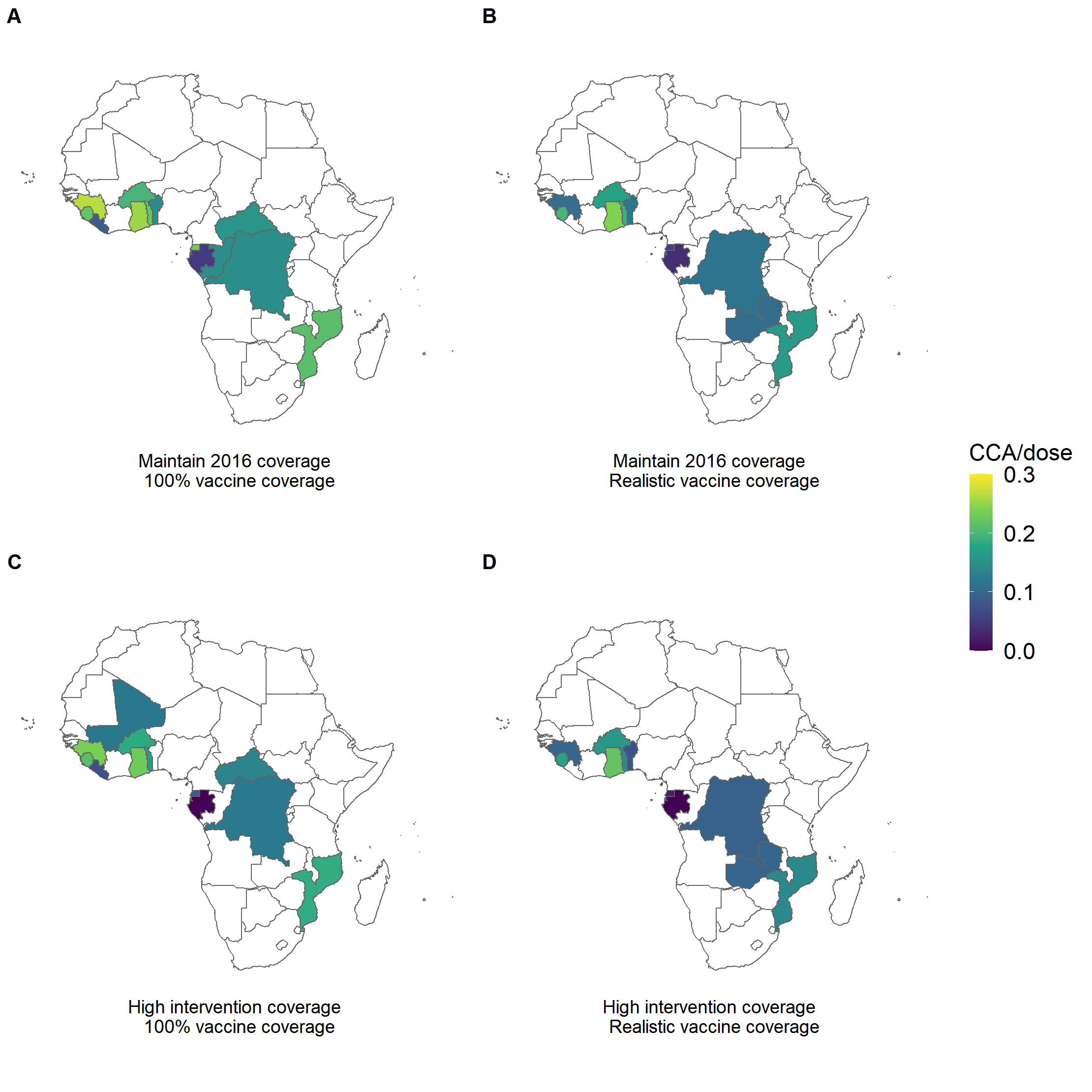

Supplement: S1 Fig — Four combinations of the baseline interventions and vaccine coverage are shown. The colour gradient represents the average annual clinical cases averted in 0- to 5-year-old children per vaccine dose (CCA/dose) in the first 5 years post-vaccine introduction. Total annual events averted are reported in Table 3. The maps were prepared using administrative boundary data from geoBoundaries [32]. CCA, clinical cases averted. (TIF) [file pmed.1003377.s008.tif]

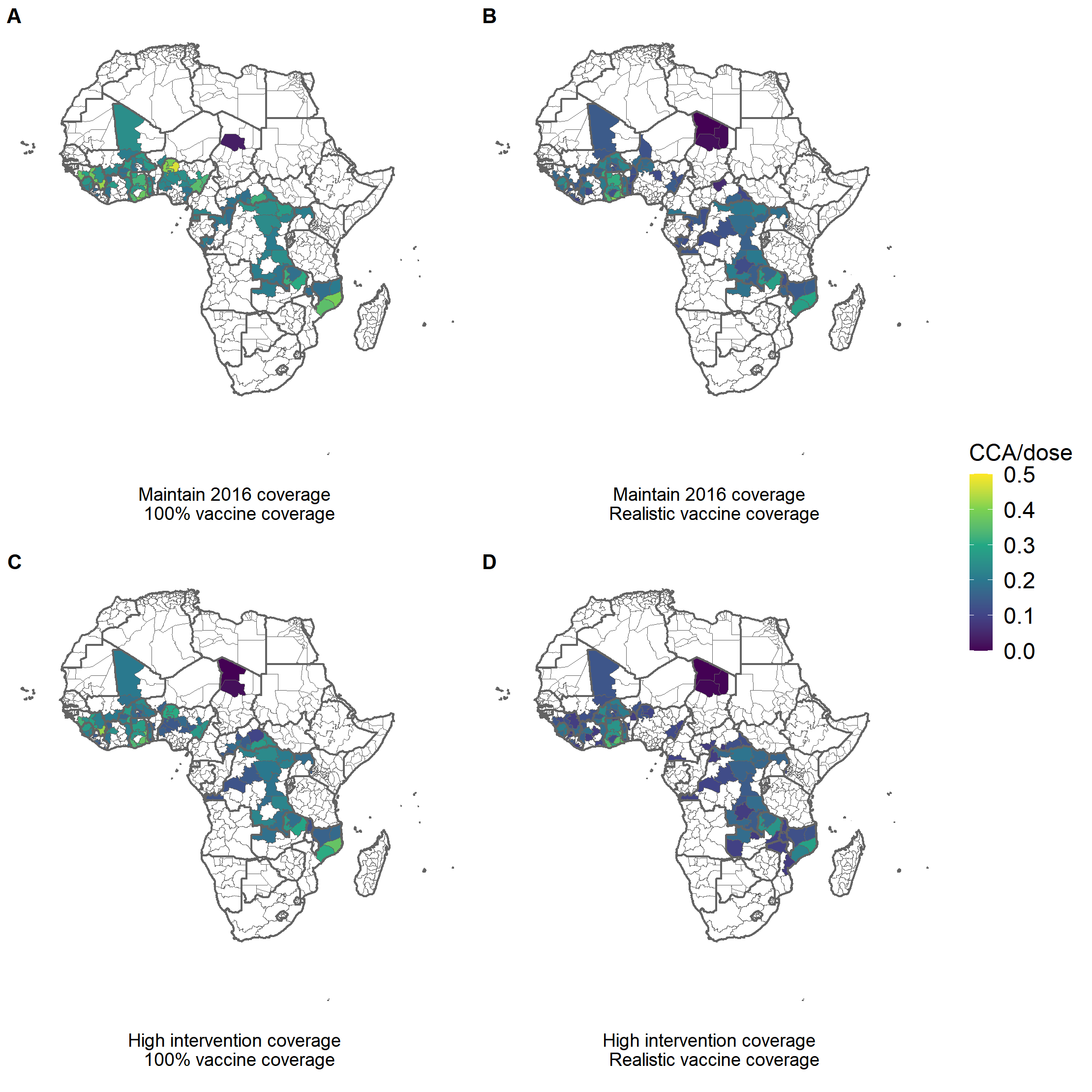

Supplement: S2 Fig — The colour gradient represents the average annual clinical cases averted in 0- to 5-year-old children per vaccine dose (CCA/dose) in the first 5 years post-vaccine introduction. Total annual events averted are reported in Table 3. The maps were prepared using administrative boundary data from geoBoundaries [32]. CCA, clinical cases averted. (TIF) [file pmed.1003377.s009.tif]

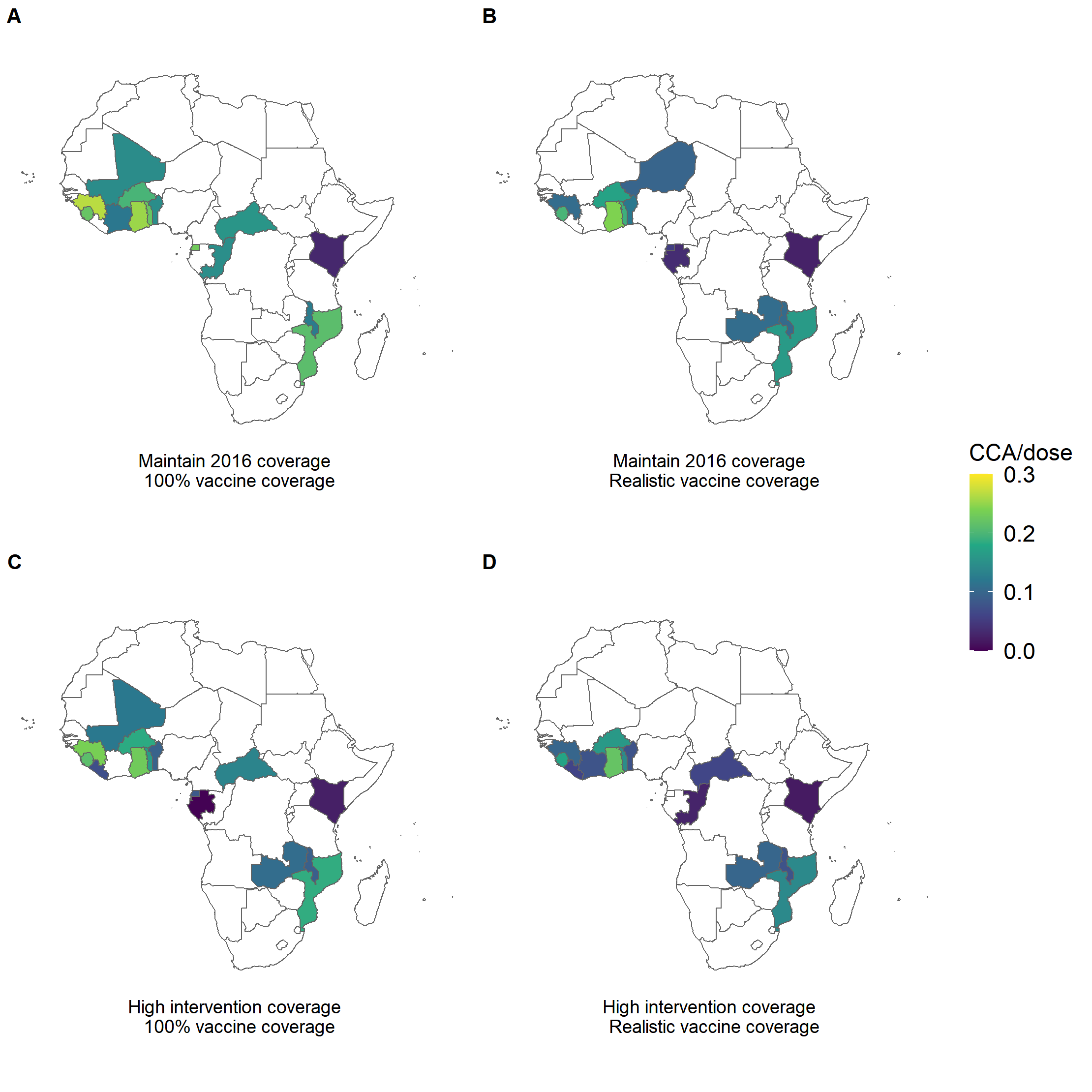

Supplement: S3 Fig — Four combinations of the baseline interventions and vaccine coverage are shown. The allocation assumes an annual constraint of 30 million vaccine doses and is based on the 4-dose schedule, with allocation performed at the country level. The colour gradient represents the average annual clinical cases averted in 0- to 5-year-old children per vaccine dose (CCA/dose) in the first 5 years post-vaccine introduction. S3 Table shows the total events averted for each scenario combination and for additional dose constraints. The maps were prepared using administrative boundary data from geoBoundaries [32]. CCA, clinical cases averted. (TIF) [file pmed.1003377.s010.tif]

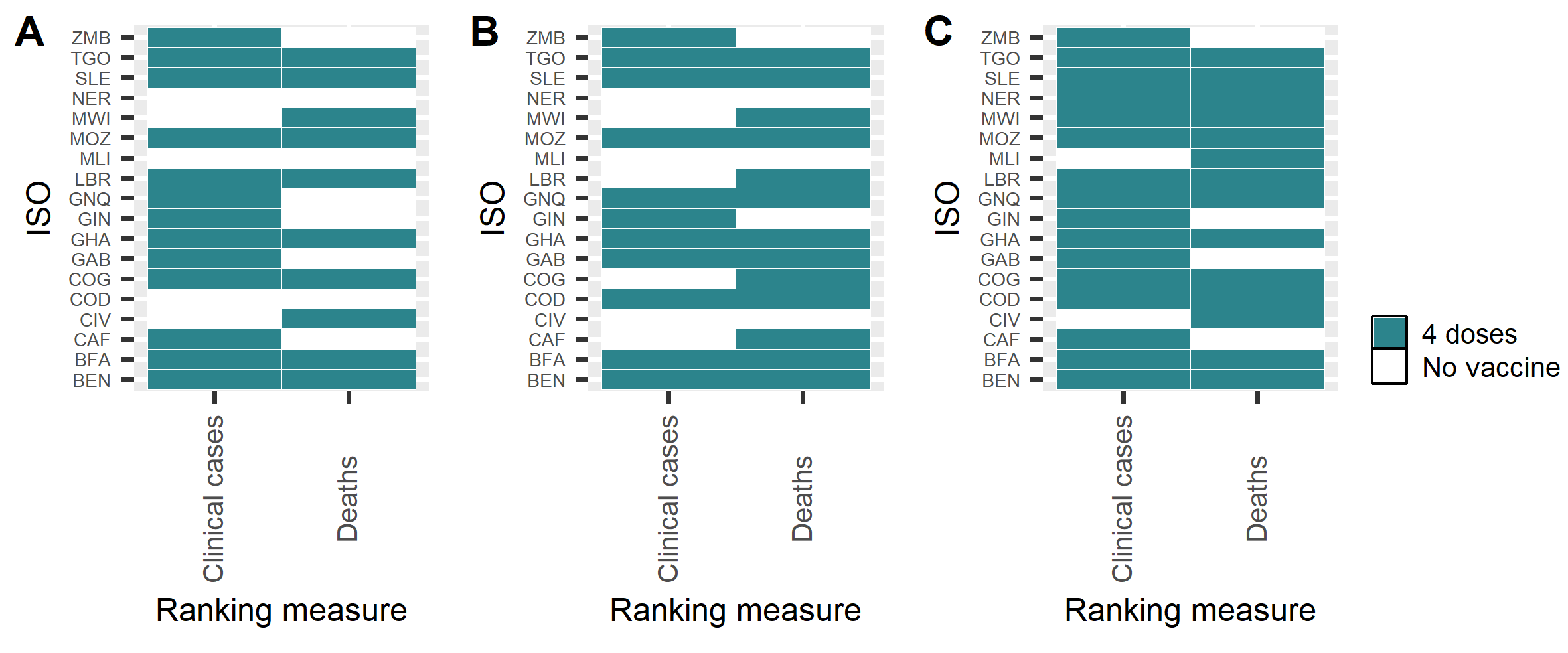

Supplement: S4 Fig — Results are shown for the “Maintain 2016 coverage” baseline intervention scenario and assuming “Realistic vaccine coverage,” for a range of annual vaccine dose constraints: (A) 20 million, (B) 30 million, and (C) 40 million. The green shading represents countries prioritised for vaccine delivery. (TIF) [file pmed.1003377.s011.tif]

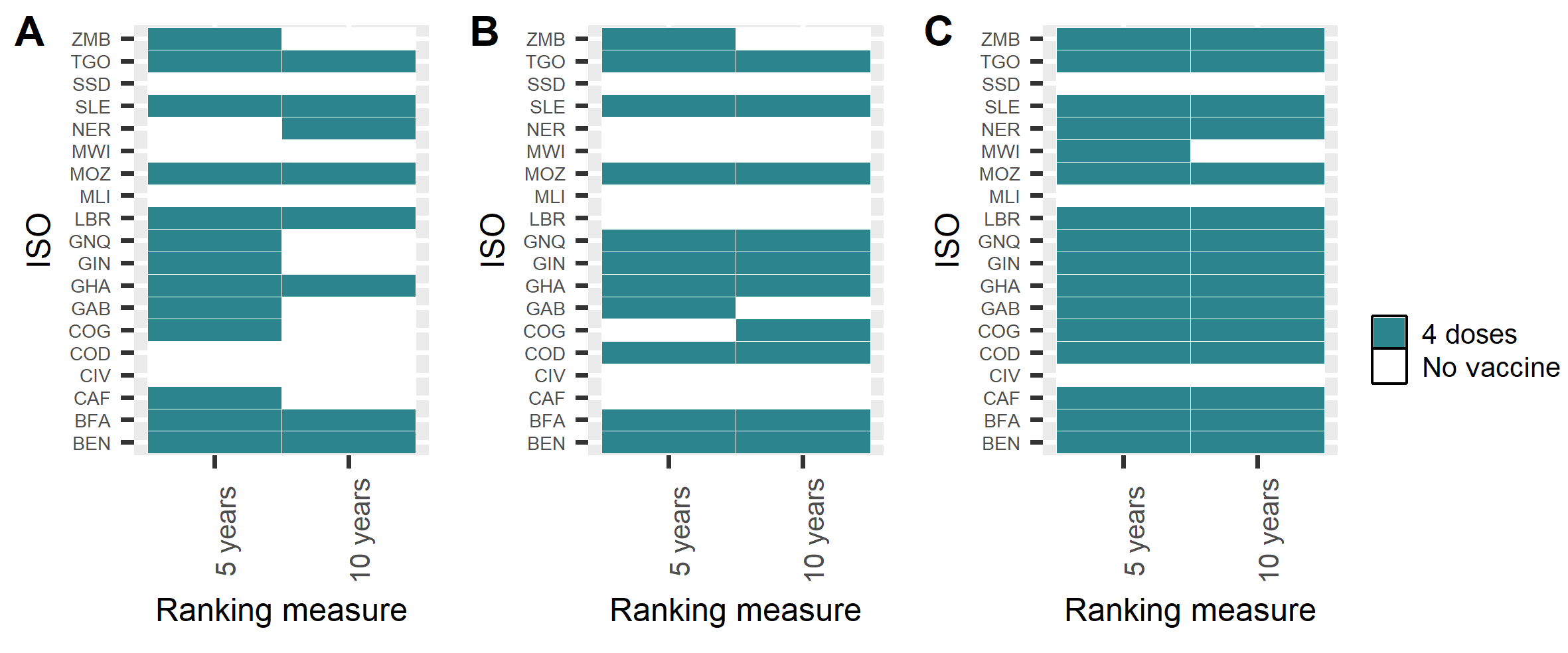

Supplement: S5 Fig — Results are shown for the “Maintain 2016 coverage” baseline intervention scenario and assuming “Realistic vaccine coverage,” for a range of annual vaccine dose constraints: (A) 20 million, (B) 30 million, and (C) 40 million. The green shading represents countries prioritised for vaccine delivery. (TIF) [file pmed.1003377.s012.tif]

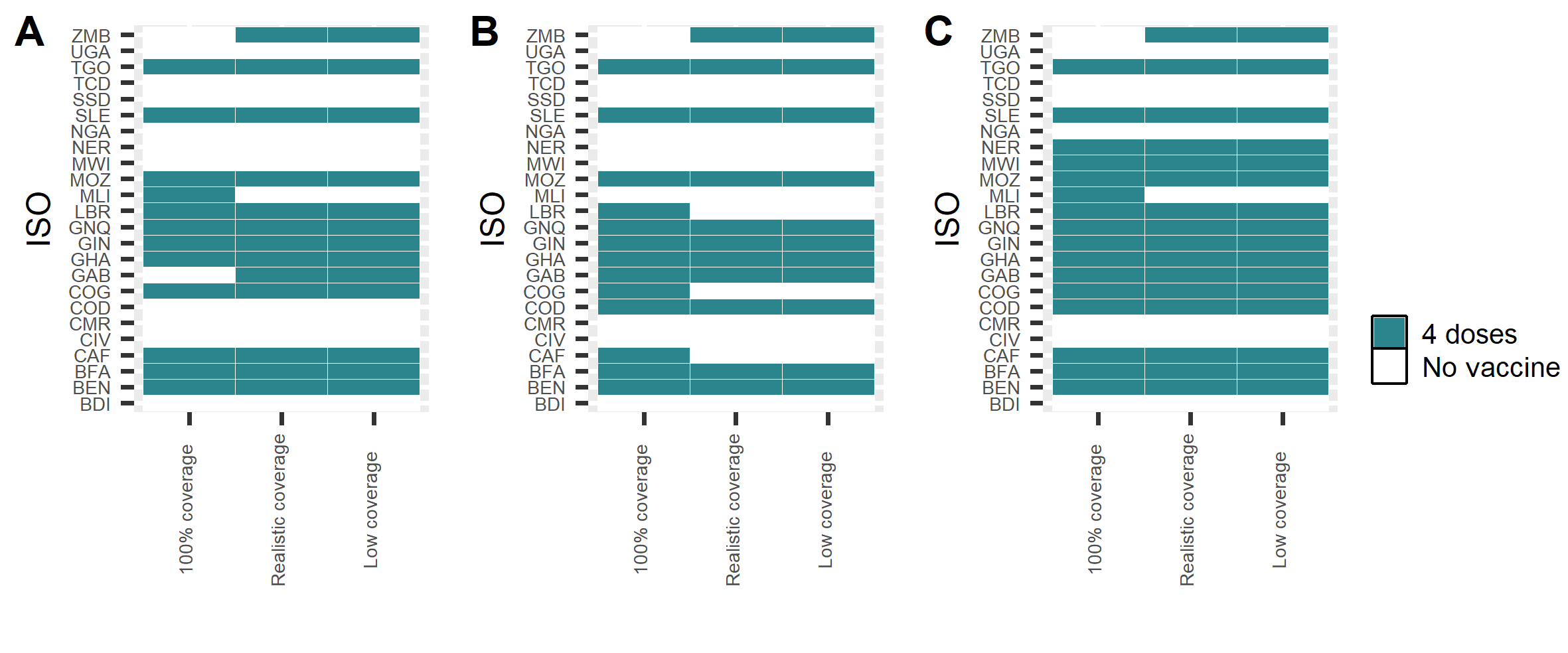

Supplement: S6 Fig — The “100% coverage” and “Realistic coverage” are as described in Table 2, and the “Low coverage” scenario assumes that coverage of doses 1–3 is set at 75% of DTP3 coverage, with coverage of dose 4 set to 80% of dose 3. Results are shown for the “Maintain 2016 coverage” baseline intervention scenario (Table 1), for a range of annual vaccine dose constraints: (A) 20 million, (B) 30 million, and (C) 40 million. The green shading represents countries prioritised for vaccine delivery. Because even with suboptimal coverage we assumed that all vaccine doses are allocated, when we modelled a vaccine take-up that is still proportional to DTP3, the country allocation did not change compared to the “Realistic coverage” scenario. DTP3, diphtheria, tetanus and pertussis vaccine dose 3. (TIF) [file pmed.1003377.s013.tif]
