# Supplementary material for: Estimated impact of RTS,S/AS01 malaria vaccine allocation strategies in sub-Saharan Africa: A modelling study
Source: PLoS Med. 2020 Nov 30;17(11):e1003377. doi: 10.1371/journal.pmed.1003377 (PMC7703928; doi:10.1371/journal.pmed.1003377)
Supplement: Attachment — Submitted filename: hogan.pdf [file pmed.1003377.s014.pdf]

This paper models allocation strategies for a vaccine against malaria.

I was asked for a statistical report and I interpret that to include all aspects of the design and conduct of the study.

Points of detail

Page 7 I think it might be helpful to call out Figure S5, and Tables S7 to S10 in the appropriate places in the description of the methods. In an ideal world they would be hyper–linked in from the text but I suspect that is not possible.

Page 11, Figure 1A I think we might have an explanation of the slightly strange behaviour of the solid blue line here between 40 and 50 million doses. Is this the same phenomenon as mentioned later where countries move in and out as the number of available doses increases?

Page 12 Thank you for providing the glossary. I thought I had quite a good grasp of African geography but GNQ had me baffled.

Figure S5 I would have expected to see an absorbing state for death. I think I can construct an explanation for why it is not needed but perhaps the authors could make it explicit. Section 2.1.4 does mention mortality.

Table S9 Is there an justification for each parameter choice to be found in references 4 and 7 to 9? If not then the other parameters need to have their source somehow. In an ideal world each row would cite its source independently.

Table S10 I think we need a reference to the source of these parameter values. I assume it is one of reference 10 or 11 which are cited earlier on.

Point of more substance

The only thing missing from this comprehensive report as far as I can see is access to the model so that other researchers can run it using different values for some of the key parameters like the number of doses and the coverage. As it stands, for instance, someone who took a more gloomy view of what a realistic scenario is would not be able to see how that would affect the clinical outcome. It would be helpful if the code could be made available

in some public repository with a DOI. I can see that an argument could be made that the model as described here is the model and anyone trying to replicate should code it up him or herself as a failure to replicate would then mean the description here is faulty but I think that is a very strong line on reproducibility of research.

Conclusion

No major issues. If people do not like the authors' conclusions the model is all there for them to do their own thing. I think we should resist the temptation to ask if the model is right as we all know that all models are wrong and the only sensible question is 'Is it useful?'.

Michael Dewey
