# Supplementary material for: Estimated impact of RTS,S/AS01 malaria vaccine allocation strategies in sub-Saharan Africa: A modelling study
Source: PLoS Med. 2020 Nov 30;17(11):e1003377. doi: 10.1371/journal.pmed.1003377 (PMC7703928; doi:10.1371/journal.pmed.1003377)
Supplement: Attachment — Submitted filename: PMEDICINE-D-20-00728R1_reviewer_comments_responses.docx [file pmed.1003377.s015.docx]

**Requests from the Editors**

Thank you for the opportunity to revise and resubmit our manuscript. We have addressed the specific requests as follows.

1. The abstract should be structured using the PLOS Medicine headings (Background, Methods and Findings, Conclusions -- Methods and Findings a single combined sub-section).

*Response:* This is now done.

2. In the Background section of the abstract, it would be good to also say something about the proven efficacy of the vaccine in trials, since the abstract just starts with the information about pilot implementation without saying anything about this being done against a background of the phase III trial data.

*Response:* We have changed the first sentence of the abstract to read:

“The RTS,S/AS01 vaccine demonstrated moderate efficacy against *P. falciparum* malaria infection in phase 3 trials, and pilot implementation of the vaccine has recently begun in three African countries.”

3. In the last sentence of the Abstract Methods and Findings section, please describe the main limitation(s) of the study's methodology.

*Response:* We have now included:

“We did not account for within-country variation in vaccine coverage, and the optimisation was based on a single outcome measure, therefore this study should be used to understand overall trends rather than guide country-specific vaccine allocation.”

We also streamlined some sentences to reduce the word count slightly.

4. At this stage, we ask that you include a short, non-technical Author Summary of your research to make findings accessible to a wide audience that includes both scientists and non-scientists. The Author Summary should immediately follow the Abstract in your revised manuscript. This text is subject to editorial change and should be distinct from the scientific abstract. Please see our author guidelines for more information: <https://journals.plos.org/plosmedicine/s/revising-your-manuscript#loc-author-summary>

*Response:* This is now included in the revised manuscript.

5. If possible, please reformat the in-text reference callouts so these are numbered citations in square brackets (eg [1, 2]) etc rather than superscript numerals. This should be quick and easy if referencing software was used. Many thanks.

*Response:* This is now done.

**Responses to reviewers' comments**

Thank you to the three reviewers for taking the time to read the manuscript thoroughly and provide constructive suggestions and comments. We have made changes based on the review comments and we believe the paper has improved. Below we have numbered each comment and addressed how the manuscript has been updated to reflect these suggestions.

Note that we have also updated S4 Figure (sensitivity to deaths as an outcome measure) to make it more interpretable, and included an additional supplementary figure, now S5 Figure, to show sensitivity to clinical cases over 10 years as an outcome measure, as we stated in the methods that we had done this but not reported it in the results section of the manuscript.

**Reviewer #1**

1. In the introduction on p5, the authors claim that the age groups for vaccination in the large-scale randomized controlled clinical trial done in 11 African sites and published in 2015 were "approximately 4, 5, 6 and 25 months". This puzzles me because the schedule in the RCT was 1,2,3, and 18 months. Since it was an RCT it was strictly enforced. The vaccination schedule at 5, 6 and 25 months is the one chosen for the pilot implementation in 3 countries in order to make it more EPI-compatible. In addition, because the vaccine is now implemented in a real-world situation, there will inevitably be some variability in the age at vaccination. Given how age-sensitive malaria incidence and mortality are, this difference must be considered. Since the vaccine parameters (especially age-efficacy) were derived from the RCT, the authors should mention this difference and have a clear explanation as to why it could matter or not. If it is likely to matter (which I believe), then an analytical approach should be designed to take that aspect into consideration.

*Response:* In the phase 3 trial there were two groups: infants aged 6-12 weeks at dose one, and children aged 5-17 months at dose one, therefore in each group there was some variability in age at vaccination in the trial. Vaccine efficacy was found to be higher in the older group. We have now clarified the age group in the methods section (page 8 para 2):

“This model captures the dynamics observed in the phase 2 and 3 trials in children aged 5–17 months at the first vaccine dose.”

In the study of immunogenicity after RTS,S vaccination, upon which the vaccine efficacy parameters for this modelling study are based, age at vaccination was considered as a determinant of immunogenicity, but the change in antibody titre following vaccination associated with a 1-month change in age was small (White et al, Lancet Inf Dis 2015, doi: 10.1016/S1473-3099(15)00239-X). As we are modelling the impact of vaccination in a real-world situation, we modelled a schedule of 6, 7.5 and 9 months for the first three doses, and 27 months for the fourth dose, because the 6 and 9 month doses align with routine health care visits. In addition, in the pilot vaccination program, the first vaccine dose is offered at 6 months of age in Ghana and Kenya, and at 5 months in Malawi. We have included these points in the methods section (page 8 para 2):

“We implemented the vaccine under a continuous EPI four-dose schedule targeted at the 5–17-month age group, in which the first three doses are administered at months 6, 7.5 and 9 and the fourth dose at month 27, such that the first and third doses may align with routine child health appointments, and that the first dose at 6 month corresponds to the first scheduled RTS,S dose for the pilot program in Ghana and Kenya (noting that in Malawi the first RTS,S dose is scheduled at 5 months).”

Therefore, we consider that we are modelling the most likely age at immunisation, and the impact of small changes to age at immunisation is likely to be small. Note that this is also the approach taken in previous work (Penny et al, Lancet, 2016, doi: 10.1016/S0140-6736(15)00725-4).

2. Modelling parameters for vaccine coverage were set to two levels: (1) current EPI levels for DPT3, and (2) 100% coverage. Informal evidence from the three pilot countries suggest that current uptake of RTS,S is much lower than for current EPI vaccines. There are a number of possible reasons for that. Without getting into a causal discussion, it seems that in addition to have a 100% coverage parameter it would be desirable to also have a scenario in which coverage is lower than the average EPI level for DPT3. 100% coverage is completely unrealistic, but that parameter value has the benefit of showing how good the vaccine could theoretically be. But to get a better sense of how valuable RTS,S would be if coverage was not as high as expected (which is now a very likely outcome of the current pilot implementation), modelling a lower uptake would be practically of great relevance.

*Response:* Thank you for this suggestion. We have included an additional sensitivity analysis to illustrate the impact on country allocation if vaccine coverage is lower than DTP3 (by setting doses 1-3 to 75% of DTP3, and dose 4 at 80% of dose 3). We have noted this extra analysis in the methods section (page 10, paragraph 2) and the new figure is now S6 Figure. We found that because even with suboptimal coverage we still assume that all doses are allocated, with lower vaccine takeup than DTP3, the country allocation does not change compared to the “Realistic coverage” scenario.

3. Discussion. No comment is made on the fact that distributing the vaccine over a maximum number of countries according to a sub-national impact optimization process is actually going to be very challenging. It is unlikely that countries will agree to such processes very easily. So while it is very good to show how this process of allocating at sub-national level can optimize limited resources, a caveat on the feasibility of this approach should be more clearly introduced in the discussion. The authors mention other GAVI-supported vaccines that are distributed according to risk areas. Giving such examples and showing possible analogies with the malaria vaccine situation would be very helpful and a good way of discussing this.

*Response:* Thank you for this suggestion. We had noted in the discussion that “any sub-national introduction will entail additional challenges with planning, logistics and public perception”. We have now provided some additional context to explain that

“given the increased spatial heterogeneity of malaria burden, there is an existing precedent for targeting other interventions for malaria control, and a broader focus on data-driven sub-national stratification of interventions within countries in order to make the most effective use of limited resources” (ref: <https://www.who.int/malaria/mpac/statements/malaria-policy-guidance/en/>) (page 14, para 1).

We have also noted that targeted vaccination takes place for diseases such as yellow fever and cholera in the context of outbreaks (page 14):

“However, targeting of vaccine delivery does occur for some vaccines where disease risk is geographically focal, such as during outbreaks of yellow fever and cholera.”

4. References start with No 10, and lower number appear later. Presumably, this should be fixed?
    *Response:* Thank you. This has now been corrected.

**Reviewer #2**

1. Page 7 I think it might be helpful to call out Figure S5, and Tables S7 to S10 in the appropriate places in the description of the methods. In an ideal world they would be hyperlinked in from the text but I suspect that is not possible.

*Response:* Figure S5 and Tables S7 to S10 (now S5 Figure and Tables A to D in S1 Appendix) have now been cross-referenced in the methods section of the main text (see pages 7 to 8).

2. Page 11, Figure 1A I think we might have an explanation of the slightly strange behaviour of the solid blue line here between 40 and 50 million doses. Is this the same phenomenon as mentioned later where countries move in and out as the number of available doses increases?

*Response:* We have now included an explanation in the text and cross-referenced Figure 1A:

“For example, moving from 40 to 45 million doses in the *100% vaccine coverage* scenario leads to the inclusion of Nigeria (and results in the steep increase in clinical cases averted in Figure 1A).”

3. Page 12 Thank you for providing the glossary. I thought I had quite a good grasp of African geography but GNQ had me baffled.

*Response:* No change needed.

4. Figure S5 I would have expected to see an absorbing state for death. I think I can construct an explanation for why it is not needed but perhaps the authors could make it explicit. Section 2.1.4 does mention mortality.

*Response:* As no other compartments/states in the model depend on the number of malaria-associated deaths, this state is not included in the model framework but rather is calculated separately as part of the post-processing. This is noted on page 18 of the supplementary methods and we have now also clarified this point in the caption to what is now Figure A in S1 Appendix.

5. Table S9 Is there a justification for each parameter choice to be found in references 4 and 7 to 9? If not then the other parameters need to have their source somehow. In an ideal world each row would cite its source independently.

*Response:* We have now included an extra column in Table S9 (now Table C in S1 Appendix) with references for each parameter, and also noting whether each parameter is fitted or fixed (S1 Appendix).

6. Table S10 I think we need a reference to the source of these parameter values. I assume it is one of reference 10 or 11 which are cited earlier on.

*Response:* We have now expanded the caption for this table (now Table D in S1 Appendix) to clarify the source of the parameter values:

“The model parameters are those reported in Table 3 of White et al.^11^ The values for the peak anti-CSP following the third and fourth dose were each calculated as the median of the 11 site values in the phase 3 RTS,S/AS01 trial.^11^”

7. The only thing missing from this comprehensive report as far as I can see is access to the model so that other researchers can run it using different values for some of the key parameters like the number of doses and the coverage. As it stands, for instance, someone who took a more gloomy view of what a realistic scenario is would not be able to see how that would affect the clinical outcome. It would be helpful if the code could be made available in some public repository with a DOI. I can see that an argument could be made that the model as described here is the model and anyone trying to replicate should code it up him or herself as a failure to replicate would then mean the description here is faulty but I think that is a very strong line on reproducibility of research.

*Response:* Thank you for this suggestion. We have now expanded the Data Availability Statement to include details of code availability:

“The individual-based model of malaria transmission is described in the Supplementary Material, and the model code is available to download at https://github.com/jamiegriffin/Malaria_simulation. Malaria transmission model outputs, code to run the ranking algorithm, and code to produce the figures and tables in the manuscript is available to download at https://github.com/mrc-ide/rtss_prioritisation.”

**Reviewer #3**

1. Abstract: "we estimate 4.3 million malaria cases and 22,000 deaths in children U5 years could be averted per year at a dose constraint of 30 million. This decreases to 3.0 million cases / 86,000 deaths at a dose constraint of 20 million" Is this a mistake? Why does the number of deaths averted decrease with fewer vaccine doses?

*Response:* Thank you for this correction. Yes, the number of deaths was incorrect in the text description (we used the number for severe cases instead of deaths, although the tables were correct). We have corrected all of the results for deaths in the abstract and checked the other in-text results. The abstract now reads:

“…we estimate 4.3 million malaria cases and 22,000 deaths in children **younger than** 5 years could be averted per year at a dose constraint of 30 million. This decreases to 3.0 million cases / **14**,000 deaths at a dose constraint of 20 million and increases to 6.6 million cases / **38**,000 deaths at a dose constraint of 60 million. At 100% vaccine coverage, these impact estimates increase to 5.2 million cases / **27**,000 deaths, 3.9 million cases / **19**,000 deaths and 10.0 million cases / **51**,000 deaths respectively. Under realistic vaccine coverage, if the vaccine is prioritised sub-nationally, 5.3 million cases and **24**,000 deaths could be averted at a dose constraint of 30 million.”

2. Perhaps something went awry with the numbering of the references because the first one is number 10.

*Response:* Thank you. This has now been corrected.

3. The authors consider two scenarios: "remain at 2016 intervention coverage or achieve WHO Global Technical Strategy targets". Given the coronavirus pandemic, it seems very possible that coverage of malaria control interventions will decrease in the coming years. I wonder if the authors should consider a scenario where coverage of malaria control interventions decreases, leading to an increase in prevalence in 2–10-year-olds.

*Response:* It is likely that the provision of malaria interventions and treatment of clinical disease will decrease as the COVID-19 pandemic take effect in sub-Saharan Africa. However, it is not clear that the impacts on malaria will be long-lasting; for example, it is possible that long-lasting insecticide-treated net campaigns will be delayed for 12 months, mitigating the immediate impact on malaria control (https://www.imperial.ac.uk/media/imperial-college/medicine/mrc-gida/2020-05-01-COVID19-Report-18.pdf). Further, given the longer time horizon for RTS,S introduction, with the pilot implementation still underway and wider rollout not expected before 2022, we believe an additional scenario would not be beneficial. No changes made.

4. Methods paragraph 2: While I think it is reasonable to relegate the details of the mathematical model to the supplementary material, the authors should include the mathematical details of how vaccine efficacy is modelled in the main text.

*Response:* We have now included details of the vaccine efficacy equation in the main text (see page 8, paragraph 2), but retained the rest of the model description in the supplementary material due to space constraints.

5. The authors say "Efficacy was assumed to occur following the third dose". It is reasonable to assume that absolutely no protection is conferred by the first 2 doses?

*Response:* We assumed that no partial protection is conferred from the initial two vaccine doses, as this is consistent with reporting from the phase 3 RTS,S/AS01 trial. We have now included this point in the Methods section (page 8, paragraph 2). However, we do acknowledge that it is possible that some protection could be conferred from the initial two doses, but given that we don’t have any information or trial data on the partial efficacy from the earlier doses, we were not able to include this impact in our model. We have now included this point as a limitation in the Discussion section:

“Second, we did not model any vaccine-induced protection until after the third dose. It is possible that some protection would be conferred from the first two doses, but additional data would be needed to capture this impact in the model.”

6. Methods paragraph 6: I think "5-17-month-old children at 6, 7.5, 9 and 27 months of age" doesn't quite make sense. 27 months is not in the range of 5-17 months. What is the significance of 5-17-month here? Should it be 6-27 month old children?

*Response:* In the phase 3 trial, the vaccinated child group were aged 5 – 17 months at first vaccine dose, therefore there was some variability in age at vaccination. We have now clarified the age group in the methods section (page 8 para 2):

“This model captures the dynamics observed in the Phase II and III trials in children aged 5–17 months at the first vaccine dose.”

As we are modelling the impact of vaccination in a real-world situation, we modelled a schedule of 6, 7.5 and 9 months for the first three doses, and 27 months for the fourth dose, because the 6 and 9 month doses align with routine health care visits. In addition, in the pilot vaccination program, the first vaccine dose is offered at 6 months of age in Ghana and Kenya, and at 5 months in Malawi. We have included both of these points in the methods section (page 8 para 2):

“We implemented the vaccine under a continuous EPI four-dose schedule targeted at the 5–17-month age group, in which the first three doses are administered at months 6, 7.5 and 9 and the fourth dose at month 27, such that the first and third doses may align with routine child health appointments, and that the first dose at 6 month corresponds to the first scheduled RTS,S dose for the pilot program in Ghana and Kenya (noting that in Malawi the first RTS,S dose is scheduled at 5 months).”

7. Methods paragraph 8. I think the way countries or regions were chosen was quite simple. Could the authors try to explain it again, because I found the paragraph somewhat opaque. Using jargon such as "steepest-descent algorithm" does not, I think, aid clarity.

*Response:* We have amended the explanation to explain the ranking algorithm more clearly, although we have retained the term “steepest-descent algorithm” as we believe it is appropriate to include the technical term (changes are underlined):

“Given a constraint in vaccine supply, we applied a steepest-descent algorithm to prioritise either countries or admin-1 units for vaccine delivery. For introduction at the sub-national level, each admin-1 unit was treated independently of country. The algorithm was applied as follows: the number of events (clinical cases or deaths) averted per vaccine dose for each intervention scenario over a given time horizon was calculated and used to rank each country or admin-1 region in descending order. Beginning with the largest events averted per dose, countries or regions were then selected until the number of total vaccine doses required would exceed the vaccine supply level.”

8. Results line 3: Should it be 157,000 rather than 157,00? If so, perhaps the authors could check the other numbers in the paragraph.

*Response:* This should be 27,000 (please see next comment).

9. The number of 157,000 deaths averted (if that is correct) does not agree with the 27000 given in table 2. I think the numbers of deaths in the text correspond to the numbers of severe cases in the table.

*Response:* Thank you for pointing out this error – yes, the results for severe cases were in the text rather than deaths. All of the in-text results of deaths averted have been checked and updated.

10. Table 2: The description of the table does not say over what timescale the deaths/cases are averted. Does it assume 4 doses?

*Response:* We have clarified in the caption to Table 2 that “The impact is the annual events averted in 0–5-year-old over five years following vaccine introduction, for a four-dose schedule.” We have also made a similar change to the captions in Tables S2–S5.

11. At the end of the first paragraph of the results, table s1 is referenced. I think the authors mean table s2.

*Response:* Thank you. This has now been corrected.

12. After multiple readings I still do not understand figure 5. Could the author please clarify? I don't understand it enough even to suggest an improvement, but one thing that is not clear to me is what is the difference between A, B and C (and likewise between D, E and F).

*Response:* We had not fully explained that the difference between A, B, and C (and D, E, and F) was different levels of coverage of the fourth vaccine dose, relative to the third dose. A, B and C represent 60%, 80%, and 100% coverage of the fourth dose respectively, relative to that of the third dose (and the same for D, E and F). We have now expanded the caption to reflect this change and updated Figure 5 itself to include a short caption on each sub-figure. We have also expanded the caption to help further explain the Figure:

**“Figure 5: Country-level vaccine allocation for different dose schedules and fourth dose coverage.** The upper row shows country allocation for a four-dose schedule only, for three levels of coverage of the fourth dose as a proportion of third dose coverage: 60%, 80% and 100% (A, B, C). The lower row shows allocation where there is the option of either a three- or four-dose schedule, for the three levels of fourth dose coverage (D, E, F). Coverage of the first three doses was based on DTP3 coverage in 2017 and the annual dose supply was limited to 30 million doses per year. The *Maintain 2016 coverage* scenario is shown, and results for the *High intervention coverage* scenario are in S6 Table.”

13. Equation 21 in supplementary material. Is "max" in the wrong place?

*Response:* Thank you. Yes, this has now been corrected.
